# Modelling compartmentalization towards elucidation and engineering of spatial organization in biochemical pathways

**DOI:** 10.1038/s41598-017-11081-8

**Published:** 2017-09-21

**Authors:** Govind Menon, Chinedu Okeke, J. Krishnan

**Affiliations:** 1 Department of Chemical Engineering, Centre for Process Systems Engineering, Imperial College London, London, SW7 2AZ UK; 2 Institute for Systems and Synthetic Biology, Imperial College London, London, SW7 2AZ UK

## Abstract

Compartmentalization is a fundamental ingredient, central to the functioning of biological systems at multiple levels. At the cellular level, compartmentalization is a key aspect of the functioning of biochemical pathways and an important element used in evolution. It is also being exploited in multiple contexts in synthetic biology. Accurate understanding of the role of compartments and designing compartmentalized systems needs reliable modelling/systems frameworks. We examine a series of building blocks of signalling and metabolic pathways with compartmental organization. We systematically analyze when compartmental ODE models can be used in these contexts, by comparing these models with detailed reaction-transport models, and establishing a correspondence between the two. We build on this to examine additional complexities associated with these pathways, and also examine sample problems in the engineering of these pathways. Our results indicate under which conditions compartmental models can and cannot be used, why this is the case, and what augmentations are needed to make them reliable and predictive. We also uncover other hidden consequences of employing compartmental models in these contexts. Or results contribute a number of insights relevant to the modelling, elucidation, and engineering of biochemical pathways with compartmentalization, at the core of systems and synthetic biology.

## Introduction

Information processing, and indeed, most vital aspects of cellular life are underpinned by complex and sophisticated protein and genetic networks. These networks have many hallmarks which distinguish them from networks in non-living contexts: they are shaped by evolution and consequently not parsimonious in construction, they contain abundant nonlinearity including feedback regulation at many levels; they make use of different information processing modules; and finally are generally robust in function^1^. In addition, due to small numbers of molecules, they are subject to stochastic fluctuations^2^. Dissecting these information processing networks, to connect their realization to their function in different contexts, is a major theme of systems biology. Engineering information processing towards various applications, or even towards a better understanding of the underlying biology is the goal of synthetic biology.

In spite of considerable strides in understanding the organization and functioning of these networks in a range of contexts, there are still a number of basic questions and issues which remain unanswered, and in some cases even unquestioned. One aspect that needs to be addressed is the spatial organization of pathways^3^. It is well known that spatial organization of pathways, with components distributed between different locations: the membrane, the cytoplasm (in different locations therein), and membrane bound compartments such as organelles, vesicles, and the nucleus, is ubiquitous in both signaling^4–7^and metabolic processes^8,9^. In fact, even bacterial cells, which lack organelles, exhibit precise spatial organization of processes^10,11^, including compartmentalization of metabolic pathways^12^. While this spatial organization is generally recognized, its effect on the functioning of the pathways is generally not well explored. Very often, it is either explicitly or implicitly assumed that spatial organization has a minor role, and can be subsumed within a purely temporal (kinetic) description of the pathways. Compartmentalization is a hallmark of cellular pathways, and there have been attempts to incorporate this aspect in models of different intracellular signalling processes^7,13,14^. In spite of this, there is still a vast chasm which needs to be bridged to effectively address this issue. In recent work, we developed a framework to understand the effect of compartmentalization on different types of canonical biochemical modification sequences and pathways^15,16^.

A considerable impetus for understanding the effect of compartmentalization comes from trying to engineer spatial organization in synthetic biology and beyond. The use of proteins and nucleic acids as synthetic scaffolds for spatial organization of enzymatic modification pathways has been realized in multiple studies^17,18^. Engineered microcompartments which localize the steps of a metabolic pathway have been suggested as a means of increasing pathway flux^19,20^. Multi-compartment lipid vesicles^21,22^ are an example of membrane bound compartments developed as a platform to regulate the functioning and behaviour of biochemical pathways. Here, particular steps of a metabolic cascade are localized in certain compartments, and the remaining steps in other compartments. The fact that only some entities can cross between compartments is an experimental basis for segregating reactions. Compartmentalization of genetic circuits in different cell free environments has also been investigated, including encapsulation by lipid vesicles^23–25^ and by compartments carved into a silicon chip^26,27^. Finally, there have also been attempts to spatio-temporally control the location of entities, using optical or magnetic means^28–30^.

In addition to the regular systems and synthetic biology motivations, compartmentalization is also of considerable interest from an evolutionary perspective^31,32^. It is clear that there is a certain degree of localization/compartmentalization in bacteria, but that this is very much greater in eukaryotes. How this new feature is exploited in different cell types and to what end is still an open question. This of course depends on the type of pathway and the type of information processing. Assessing compartmentalization from this angle may provide vital clues into why pathways are organized the way they are. Finally, there are experimental studies which explore the origins of life, using vesicles to compartmentalize reactions and thereby capture the essential features of proto-cells.

Taken together, it is clear that compartmentalization is ubiquitous in natural systems, a key ingredient in evolution, and a very promising tool for engineering biochemical pathways in cells and cell free environments. Ultimately a clear understanding of the role of compartmentalization and an effective basis for engineering them, needs an appropriate modelling and systems framework. The choice of the modelling framework dictates what kinds of conclusions can be drawn, and whether they are sound and generalizable. Compartmental ODE models are a widespread framework for analyzing compartmental systems in the broadest sense. Even in studying compartmentalization, they are a natural choice as they allow for the modelling framework to focus on the key underlying and measurable features.

In this paper, our goal is two-fold. On the one hand we investigate the effects of compartmentalization in a series of typical biochemical pathways. This includes building blocks for both signalling and metabolic pathways, and extensions thereof. On the other hand, we investigate the most appropriate modelling framework for describing these compartmental systems, and whether compartmental ODE models provide realistic depictions as well as reliable conclusions. Through a series of investigations we establish under which conditions these comparmentalized pathways can be described by compartmental models. Our study is relevant in both systems and synthetic biology. In systems biology the fact that pathways are compartmentalized is increasingly recognized. However, models in systems biology at all levels need simplifications, to focus on the key underlying aspects. We address when and if compartmental models can reasonably capture the effects of compartmentalization, and make sound conclusions and reliable predictions therefrom. In synthetic biology, the design of compartments and manipulation of pathways need sound and robust platforms for design, analysis and engineering. In this regard the choice of modelling framework must enable this in a transparent way, while respecting the underlying physics and chemistry of the process.

This paper is organized as follows. In the next section we outline the models we use and the approach we take. We then present various results of our study. We conclude with a summary and discussion of the results. Analytical results supporting the computations are presented in the Supplementary Information.

## Models

In this section, we first describe the essential features of the compartmentalized biochemical reaction pathways that are of interest in this study. Next, we present the basic PDE framework that we use to model these systems, and describe the general form of the corresponding compartmental ODE descriptions. Following this, we discuss the criteria that we use to examine to what extent a compartmental ODE model is consistent with the system it attempts to describe.

### Compartmentalized pathways with diffusive transport

The kinds of compartmentalized biochemical pathways of interest in our study share the following features:

- A spatial domain that consists of compartments separated by intermediate spaces.
- Reactions confined to one or more compartments (realised through the compartmentalization of corresponding enzymes or substrates).
- Transport of species between compartments by diffusion.
- No chemical reactions in the intermediate spaces.

The purpose of our study is to examine the capabilities of compartmental ODE models, as tools for understanding such compartmentalized systems of biochemical pathways. PDE descriptions of these systems form a reference point.

For simplicity, we restrict ourselves to a 1-D PDE framework, and assume a single diffusivity for each species in the entire domain. However, as we explain later, most of our insights continue to be relevant to systems in 2-D and 3-D.

### 1-D PDE description

For simplicity, we consider a system with two compartments. The framework is easily extended to systems involving more compartments. For a system with *n* species, the PDE description consists of a set of coupled reaction-diffusion equations, with the following general form:

In compartment $$\mathrm{1(}\theta \in {{\rm{\Omega }}}_{1})$$,

1 $$\frac{\partial {X}_{j}}{\partial t}={f}_{j1}({X}_{1},{X}_{2}\mathrm{,...},\,{X}_{n})+{D}_{j}\frac{{\partial }^{2}{X}_{j}}{\partial {\theta }^{2}}$$

where *j* = 1, 2, …, *n*

Between comparments $$(\theta \in {{\rm{\Omega }}}_{12})$$,

$$\frac{\partial {X}_{j}}{\partial t}={D}_{j}\frac{{\partial }^{2}{X}_{j}}{\partial {\theta }^{2}}$$

In compartment $$2\,(\theta \in {{\rm{\Omega }}}_{2})$$,

2 $$\frac{\partial {X}_{j}}{\partial t}={f}_{j2}({X}_{1},{X}_{2}\mathrm{,...,}\,{X}_{n})+{D}_{j}\frac{{\partial }^{2}{X}_{j}}{\partial {\theta }^{2}}$$

Here, *θ* represents the spatial variable and *t* represents time. *X*_*j*_ represents the concentration of species *j*, and *D*_*j*_ represents its diffusivity. The spatial domain Ω is partitioned into 3 intervals - Ω_1_ and Ω_2_ representing the compartments, and Ω_12_ representing the intermediate space between compartments. No-flux boundary conditions are used at the outer edges of the domain.

The function ${f}_{j1}({X}_{1},{X}_{2},\mathrm{...},{X}_{n})$ represents the reaction kinetic terms for species *j*, associated with the reactions occurring in compartment 1, and similarly for the function ${f}_{j2}({X}_{1},{X}_{2},\mathrm{...},{X}_{n})$. If all the reactions are described by mass-action kinetics of first-order, the functions *f*_*j*_ will be linear. When the system involves closed steps, as is common in biochemical pathways, the PDE description implies a conservation of species. In such cases, the total amount of a conserved species needs to be specified when solving for the steady states of the PDE.

The different biochemical pathways examined in this study, are described below (for a two compartment scenario). Readers not interested in the details may skip directly to the section on compartmental ODE models.

#### Simple closed system: Single modification cycle

**Figure 1:**
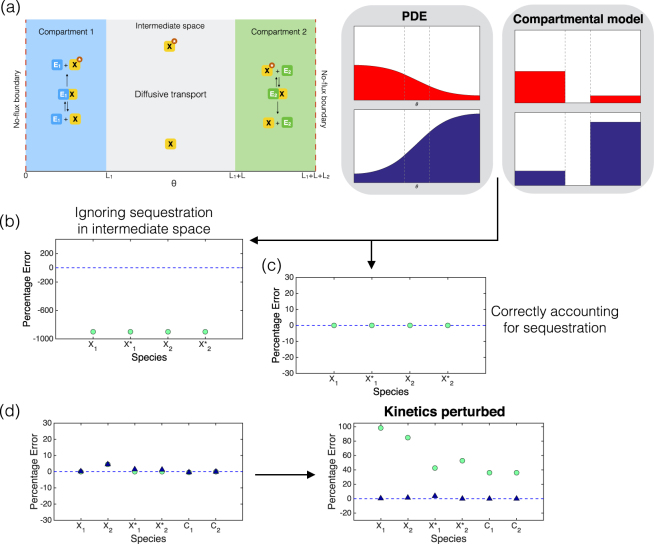
Compartmentalized single modification cycle. **(a)** Schematic for the compartmentalized pathway. The PDE description yields steady state concentration profiles of *X* and X*. The compartmental ODE gives steady state compartmental concentrations of *X* and X*. We examine the error between the PDE compartmental averages and the ODE steady state. **(b)** Percentage error for each species, between the steady state compartmental averages of the PDE model, and the steady state of a compartmental model using the same total amount of substrate as the PDE model. We consider a system with two compartments, each occupying 5 percent of the whole domain. **(c)** In the mass action regime, if the total amount of substrate in the compartments is exactly accounted for, transport parameters can be computed, which result in an exact match. **(d)** In the non-mass action regime, an exact fit cannot be obtained, even if the total amount of substrate in the compartments is accounted for. Errors are shown for both the basic compartmental model calibrated with the exact total amount of substrate in the compartments under basal conditions - green circles, and the compartmental model with modified conservation to account for amount of substrate in the intermediate space – blue triangles. For the basal kinetics, both cases match the PDE closely. When the kinetic parameter is changed (phosphatase concentration in compartment 2 increases), the modified model (blue triangles) continues to give a good fit, while the basic compartmental model with calibration under basal conditions (green circles) produces large errors.

This is a closed system consisting of a single enzymatic modification cycle involving species *X* and X*. Both species diffuse across the whole domain, and may have different diffusivites. *E*_1_, the enzyme catalyzing *X* → X* is non-diffusing and uniformly distributed in compartment 1 (see Fig. 1(a)). *E*_2_, the enzyme catalyzing X* → *X* is non-diffusing and uniformly distributed in compartment 2. The enzyme substrate complexes, *XE*_1_ and *X* ^*^ *E*_2_ (referred to as *C*_1_ and *C*_2_) are also non-diffusing. The total amount of substrate within the whole domain, consisting of free substrate (*X* and X*) and the complexes, is conserved.

**PDE model:** In compartment 1 (0 ≤ *θ* ≤ *L*_1_),

$$\begin{array}{rcl}\frac{\partial [X]}{\partial t} & = & -\,{k}_{1}[X][{E}_{1}]+{k}_{-1}[{C}_{1}]+{D}_{X}\frac{{\partial }^{2}[X]}{\partial {\theta }^{2}}\\ \frac{\partial [{X}^{\ast }]}{\partial t} & = & {k}_{2}[{C}_{1}]+{D}_{{X}^{\ast }}\frac{{\partial }^{2}[{X}^{\ast }]}{\partial {\theta }^{2}}\\ \frac{\partial [{C}_{1}]}{\partial t} & = & {k}_{1}[X][{E}_{1}]-{k}_{-1}[{C}_{1}]-{k}_{2}[{C}_{1}]\end{array}$$

Between the compartments (*L*_1_ ≤ *θ* ≤ *L*_1_ + *L*),

$$\begin{array}{rcl}\frac{\partial [X]}{\partial t} & = & {D}_{X}\frac{{\partial }^{2}[X]}{\partial {\theta }^{2}}\\ \frac{\partial [{X}^{\ast }]}{\partial t} & = & {D}_{{X}^{\ast }}\frac{{\partial }^{2}[{X}^{\ast }]}{\partial {\theta }^{2}}\end{array}$$

In compartment 2 (*L*_1_ + *L* ≤ *θ* ≤ *L*_1_ + *L* + *L*_2_ = *l*),

$$\begin{array}{rcl}\frac{\partial [X]}{\partial t} & = & {k}_{4}[{C}_{2}]+{D}_{X}\frac{{\partial }^{2}[X]}{\partial {\theta }^{2}}\\ \frac{\partial [{X}^{\ast }]}{\partial t} & = & -\,{k}_{3}[{X}^{\ast }][{E}_{2}]+{k}_{-3}[{C}_{2}]+{D}_{{X}^{\ast }}\frac{{\partial }^{2}[{X}^{\ast }]}{\partial {\theta }^{2}}\\ \frac{\partial [{C}_{2}]}{\partial t} & = & {k}_{3}[{X}^{\ast }][{E}_{2}]-{k}_{-3}[{C}_{2}]-{k}_{4}[{C}_{2}]\end{array}$$

These result in the conservation equations,

$$\begin{array}{rcl}{X}^{Total} & = & (\langle [X]\rangle +\langle [{X}^{\ast }]\rangle )l+{\langle [{C}_{1}]\rangle }_{1}{L}_{1}+{\langle [{C}_{2}]\rangle }_{2}{L}_{2},\\ {E}_{1}^{Total} & = & ({\langle [{E}_{1}]\rangle }_{1}+{\langle [{C}_{1}]\rangle }_{1}){L}_{1}\\ {E}_{2}^{Total} & = & ({\langle [{E}_{2}]\rangle }_{2}+{\langle [{C}_{2}]\rangle }_{2}){L}_{2}\end{array}$$

where $\langle \cdot \rangle$ represents the spatial average over the whole domain, and ${\langle \cdot \rangle }_{i}$ represents the spatial average over the compartment *i*.

#### Simple open system

This is an open system involving a single species *X* that is produced in compartment 1 and degraded in both compartments 1 and 2.

**PDE model:** In compartment 1 (0 ≤ *θ* ≤ *L*_1_),

$$\frac{\partial [X]}{\partial t}={k}_{0}-{k}_{1}[X]+{D}_{X}\frac{{\partial }^{2}[X]}{\partial {\theta }^{2}}$$

Between the compartments (*L*_1_ ≤ *θ* ≤ *L*_1_ + *L*),

$$\frac{\partial [X]}{\partial t}={D}_{X}\frac{{\partial }^{2}[X]}{\partial {\theta }^{2}}$$

In compartment 2 (*L*_1_ + *L* ≤ *θ* ≤ *L*_1_ + *L* + *L*_2_ = *l*),

$$\frac{\partial [X]}{\partial t}=-{k}_{2}[X]+{D}_{X}\frac{{\partial }^{2}[X]}{\partial {\theta }^{2}}$$

#### Two-site modification

This is a closed system involving interconversion between the species *X* and its modified forms, X* and X**, with the three forms belonging to a conserved pool. As in the previous models, both enzymes and complexes are non-diffusing. Enzyme *E*_1_, uniformly distributed in compartment 1, catalyzes both the forward steps *X* → X* and X* → X**, while *E*_2_, uniformly distributed in compartment 2, catalyzes both the reverse steps.

**PDE model:** In compartment 1 $(0\le \theta \le {L}_{1})$,

$$\begin{array}{rcl}\frac{\partial [X]}{\partial t} & = & -\,{k}_{1}[X][{E}_{1}]+{k}_{-1}[{C}_{1}]+{D}_{X}\frac{{\partial }^{2}[X]}{\partial {\theta }^{2}}\\ \frac{\partial [{X}^{\ast }]}{\partial t} & = & -\,{k}_{3}[{X}^{\ast }][{E}_{1}]+{k}_{-3}[{C}_{2}]+{k}_{2}[{C}_{1}]+{D}_{{X}^{\ast }}\frac{{\partial }^{2}[{X}^{\ast }]}{\partial {\theta }^{2}}\\ \frac{\partial [{X}^{\ast \ast }]}{\partial t} & = & {k}_{4}[{C}_{2}]+{D}_{{X}^{\ast \ast }}\frac{{\partial }^{2}[{X}^{\ast \ast }]}{\partial {\theta }^{2}}\\ \frac{\partial [{C}_{1}]}{\partial t} & = & {k}_{1}[X][{E}_{1}]-{k}_{-1}[{C}_{1}]-{k}_{2}[{C}_{1}]\\ \frac{\partial [{C}_{2}]}{\partial t} & = & {k}_{3}[{X}^{\ast }][{E}_{1}]-{k}_{-3}[{C}_{2}]-{k}_{4}[{C}_{2}]\end{array}$$

Between the compartments (${L}_{1}\le \theta \le {L}_{1}+L$),

$$\begin{array}{rcl}\frac{\partial [X]}{\partial t} & = & {D}_{X}\frac{{\partial }^{2}[X]}{\partial {\theta }^{2}}\\ \frac{\partial [{X}^{\ast }]}{\partial t} & = & {D}_{{X}^{\ast }}\frac{{\partial }^{2}[{X}^{\ast }]}{\partial {\theta }^{2}}\\ \frac{\partial [{X}^{\ast \ast }]}{\partial t} & = & {D}_{{X}^{\ast \ast }}\frac{{\partial }^{2}[{X}^{\ast \ast }]}{\partial {\theta }^{2}}\end{array}$$

In compartment 2 ($${L}_{1}+L\le \theta \le {L}_{1}+L+{L}_{2}=l$$),

$$\begin{array}{rcl}\frac{\partial [X]}{\partial t} & = & {k}_{8}[{C}_{4}]+{D}_{X}\frac{{\partial }^{2}[X]}{\partial {\theta }^{2}}\\ \frac{\partial [{X}^{\ast }]}{\partial t} & = & -\,{k}_{7}[{X}^{\ast }][{E}_{2}]+{k}_{-7}[{C}_{4}]+{k}_{6}[{C}_{3}]+{D}_{{X}^{\ast }}\frac{{\partial }^{2}[{X}^{\ast }]}{\partial {\theta }^{2}}\\ \frac{\partial [{X}^{\ast \ast }]}{\partial t} & = & -\,{k}_{5}[{X}^{\ast \ast }][{E}_{2}]+{k}_{-5}[{C}_{3}]+{D}_{{X}^{\ast \ast }}\frac{{\partial }^{2}[{X}^{\ast \ast }]}{\partial {\theta }^{2}}\\ \frac{\partial [{C}_{3}]}{\partial t} & = & {k}_{5}[{X}^{\ast \ast }][{E}_{2}]-{k}_{-5}[{C}_{3}]-{k}_{6}[{C}_{3}]\\ \frac{\partial [{C}_{4}]}{\partial t} & = & {k}_{7}[{X}^{\ast }][{E}_{2}]-{k}_{-7}[{C}_{4}]-{k}_{8}[{C}_{4}]\end{array}$$

These result in the conservation equations,

$$\begin{array}{rcl}{X}^{Total} & = & (\langle [X]\rangle +\langle [{X}^{\ast }]\rangle +\langle [{X}^{\ast \ast }]\rangle )l+{\langle [{C}_{1}]\rangle }_{1}{L}_{1}+{\langle [{C}_{2}]\rangle }_{1}{L}_{1}+{\langle [{C}_{3}]\rangle }_{2}{L}_{2}+{\langle [{C}_{4}]\rangle }_{2}{L}_{2},\\ {E}_{1}^{Total} & = & ({\langle [{E}_{1}]\rangle }_{1}+{\langle [{C}_{1}]\rangle }_{1}+{\langle [{C}_{2}]\rangle }_{1}){L}_{1}\\ {E}_{2}^{Total} & = & ({\langle [{E}_{2}]\rangle }_{2}+{\langle [{C}_{3}]\rangle }_{2}+{\langle [{C}_{4}]\rangle }_{2}){L}_{2}\end{array}$$

### Compartmental ODE models

A compartmental ODE description conceptualizes systems of the above form as two compartments with species transported between them. It represents diffusive transport of species between compartments using a linear term, in which the difference in species concentrations between compartments is multiplied by a proportionality constant (assumed positive). The proportionality constant for the transport from compartment 1 to 2 is the associated transport parameter. The compartmental ODE model corresponding to the system described by equations (1), takes the following form:

For compartments 1 and 2,

3 $$\begin{array}{rcl}{V}_{1}\frac{d{X}_{j1}}{dt} & = & {V}_{1}{f}_{j1}({X}_{11},{X}_{21}\mathrm{,...,}\,{X}_{n1})-{V}_{1}t{r}_{1\to 2}^{j}{X}_{j1}+{V}_{2}t{r}_{2\to 1}^{j}{X}_{j2}\\ {V}_{2}\frac{d{X}_{j2}}{dt} & = & {V}_{2}{f}_{j2}({X}_{12},{X}_{22}\mathrm{,...,}\,{X}_{n2})-{V}_{2}t{r}_{2\to 1}^{j}{X}_{j2}+{V}_{1}t{r}_{1\to 2}^{j}{X}_{j1}\end{array}$$

where *V*_*i*_ represents the volume of compartment *i*(*i* = 1,2) and the variables *X*_*ji*_ represent the concentration of species *j* in compartment *i*. $t{r}_{1\to 2}^{j}$ is the rate constant associated with the transport of species *j* from compartment 1 to compartment 2. The reaction kinetic terms, represented by the functions ${f}_{ji}({X}_{1i},{X}_{2i}\mathrm{,...,}\,{X}_{ni})$, are the same as those in the PDE model. As in the case of the PDE description, for closed steps, the compartmental model implies a conservation condition, and the corresponding total amounts need to be specified when solving for the steady states of the compartmental ODE.

The corresponding compartmental ODE models, for the different pathways considered in this study, are presented in the Supplementary Information.

### Evaluating compartmental ODE models

We examine compartmental ODEs to understand whether they can consistently describe systems of the form (1). We will primarily focus on steady state behaviour since this is the primary aspect of interest and is a natural first step of examination. In general, we can expect a certain mismatch in dynamic variation because of the differences in the way the transport is described.

We examine the quantitative correspondence between the ODE model and the PDE description, within the compartments, at steady state. We look for a quantitative match between steady-state species concentrations in the compartments in the compartmental ODE model, and the steady-state compartmental averages in the PDE description.

In any given instance, the effectiveness of a compartmental model is indicated by the degree to which the ODE steady-state matches the PDE spatial averages in the relevant compartments. One way of quantifying this is using a relative error, calculated as:

4 $${e}_{{X}_{ji}}=\frac{|{X}_{ji}^{ss}-{\langle {X}_{j}^{ss}\rangle }_{i}|}{{\langle {X}_{j}^{ss}\rangle }_{i}},$$

which gives the relative error produced by the compartmental ODE in describing the steady state concentration of species *j* in compartment *i*. Here, ${X}_{ji}^{ss}$ is the steady-state concentration of species *j* in compartment *i*, as given by the ODE, and ${\langle {X}_{j}^{ss}\rangle }_{i}$ is the steady-state spatial average of the concentration *X*_*j*_ in compartment *i*, as given by the PDE.

We write the equations in dimensionless form. We choose representative parameters which are sufficient for the purposes of our study. While quantitative results do depend on parameters, the main underlying conclusions do not, and are valid across multiple parameter sets. In this regard, we note the following points. (i) We have examined multiple regimes of enzymatic reactions, both mass action (no complex formation) and non-mass action (with enzyme-substrate complexes). (ii) We span a range of compartmental sizes, relative to the size of the domain. (iii) We examine a range of diffusion coefficients. We note that when the diffusivity becomes large, the concentration of diffusing species is essentially the same in both compartments. In such a special case, it may be possible to do without a compartmental description. (iv) We use analytical work to explain and consolidate these conclusions. Where possible, we use analytical solutions to explain the parameter dependence transparently. This is sufficient for the nature of the conclusions that we draw.

The PDE models are numerically solved in MATLAB (using ode15s), using a finite difference scheme, to find the steady-state concentration profiles of the different species. Spatial averages within the compartments and the intermediate space are then computed using a trapezoidal method. For the single modification cycle, the simple open pathway, and the two-site modification, analytical solutions are obtained in the limit where all the enzymatic reactions follow mass-action kinetics (see Supplementary Information).

## Results

Compartmentalized reaction systems are routinely encountered in biology, and compartmental ODE models are used to describe such systems. In such settings, the reactions and their consequences are the main point of interest. Such models keep the focus on the compartments where reactions occur, by describing inter-compartmental transport in a simplified way (and focussing on average concentrations in the compartments). However, movement of species between compartments occurs through transport (diffusion, and other modes). This therefore brings up a number of questions. (i) When is a compartmental ODE model an accurate and reliable description of such a compartmentalized reaction scenario? (ii) How well does it work for closed reaction systems (as seen in signalling, with conservation of species) and open reaction systems (as seen in metabolism, which involves production and degradation of species). (iii) Does this change depending on the kinetic regime of reactions (mass-action regime, non mass action regime)? (iv) How do factors such as the relative sizes of the compartments and the intervening space affect this? (v) What consequence does this have for compartmentalized reactions in systems and synthetic biology, noting both the myriad additional complexities present in cellular systems, and potentially broad types of new designs in synthetic biology? Addressing such questions demands a systematic systems investigation, which we undertake below.

Our goal is to investigate compartmentalization in canonical building blocks of signalling and metabolic pathways, focussing on when and to what extent compartmental ODE models are adequate for this. We present the investigation in three stages: (i) investigation of simple open and closed pathways, (ii) investigation of more complex closed pathways, which are building blocks for signalling cascades, and (iii) an extension of (i) to examine a basic design problem involving compartmentalization of metabolic reactions. In all these cases, we consider reactions compartmentalized in two compartments and employ PDE and compartmental ODE models as described above.

We note that, for any biochemical pathway that we study, the kinetics and compartment sizes are common to both types of models. With regard to transport, diffusion is explicitly described in the PDE model, while the compartmental model has transport parameters. For each species, there are two transport parameters in principle, describing transport between the compartments in both directions. As employed in the literature, these transport parameters are equal if the volumes of the compartments are equal. We will examine this transport parameter in our analysis.

### Simple open and closed systems

In this subsection, we focus on simple reactions (both closed and open) which are building blocks for signalling and metabolic pathways. We consider (i) A covalent modification cycle and (ii) A single reaction with production and degradation. In each case, reactions are compartmentalized. We wish to assess compartmental ODE models of such systems. In so doing, we span a range of compartment sizes (relative to the intervening space), from the thin compartment regime (small compartment size relative to intervening space) to the non-thin compartment regime. Spanning this entire range allows us to tease out multiple factors at play. We also examine the consequences of kinetic regimes, such as a mass-action kinetic regime and a non-mass action kinetic regime. Finally, since the goal of a model is to be able to make new predictions and provide new understanding, we examine the role of kinetic parameters in assessing the reliability of compartmental ODE models.

#### Simple closed systems

We start by examining a sample closed system: a covalent modification cycle, with phosphorylation and dephosphorylation in two compartments, with modified and unmodified species diffusing between compartments (see Fig. 1). Such scenarios are seen in multiple cellular contexts, including bacterial pathways where the two compartments represent the two poles of the bacteria^11^. We start the system with a specified amount of kinase and phosphatase in the respective compartments, and (for specificity) all the substrate in one of the compartments. For simplicity we assume mass-action kinetics of the enzymatic modification, and examine other cases subsequently. Appropriate transport parameters for the compartmental models are chosen (discussed in detail later). We wait till the system reaches a steady state. As seen in Fig. 1(b), there is a substantial mismatch between the steady state of the compartmental model and steady state average concentrations of the PDE.

A careful examination of both models indicates why this is the case. In the PDE model, both substrates are present not only in the compartments, but also in the intervening space. Substrates are subject to a conservation condition (since there is no *de novo* production or degradation of substrate in the relevant timescales), and this is implicit in both models. However, the compartmental model does not accurately describe the conservation of substrate. This is the reason for the mismatch. The greater the separation between compartments, the greater the mismatch in general.

#### Populating the compartmental model with correct total species amounts

Noting the source of the disparity between the models, there are different ways to reduce or remove this disparity. One way is to populate the compartmental model with a total amount of substrate which matches the total amount of substrate in the two compartments in the PDE solution. For instance, this information could be obtained from an experiment and used in the compartmental model. As shown in Fig. 1(c), using this approach (and an appropriate choice of transport parameters), we are able to obtain an exact match. Now if the modification kinetics are not mass-action, the same approach can be used. Here, for fundamental reasons, the compartmental model can never exactly match the PDE model: this is because of the presence of complexes and the associated nonlinearity in the kinetics. The presence of nonlinear kinetics necessarily implies that compartment averages of substrate and complex concentrations in the PDE cannot match that of the compartmental models. This is discussed analytically in the Supplementary Information. Indeed, changing kinetics of modification away from mass action (keeping transport parameters fixed) does lead to a degree of mismatch. This implies, that the best one can achieve with a compartmental model, is to reduce this mismatch and get as close as possible to the PDE model (for average species concentrations). The only flexibility in the compartmental model resides in the transport parameters. We can vary the transport parameters of the modified and unmodified substrate to see how close a match can be obtained. Numerically minimizing the error (see Supplementary Information) indicates that there are transport parameters which can give a very small error (discussed later). In multiple cases considered, the transport parameters for the species, happen to be in the ratio of their diffusivities. Further aspects about transport parameters are discussed later.

#### Lack of kinetic robustness

Now if we use the above approach to populate the compartmental model with the correct total amount of substrate from the PDE model (exact fit for mass action kinetics, inexact for non-mass action kinetics), we can use this compartmental model in a predictive way. We examine the response of the model to a change of kinetic parameters, which would be a natural use of such a model. How good are the predictions? We find that there is an increasing error between the compartmental model prediction and the PDE model, as the phosphatase concentration is changed from basal levels (Fig. 1(d): green circles). This suggests that even with the correct total amount of substrate, compartmental models may be subject to non-trivial levels of error when used predictively. We refer to this as kinetic non-robustness of the compartmental model. It is instructive to examine the reasons for this error. To do this, we examine two specific regimes.

#### Thin compartment: The effect of sequestration

If the size of the compartment is small relative to the size of the domain, then the transport parameters are determined as *D*/*LL*_1_ where *D* is the diffusivity of the species, *L* is the length of the intervening space and *L*_1_ the size of the compartment (see schematic in Fig. 1, and analytical discussion in Supplementary Information). With regard to sequestration, we note that the total amount of substrate in the compartments can, in general, vary with the kinetic parameters. Therefore, the assumption of a fixed total amount of substrate in the two compartments will contribute increasing errors when kinetic parameters are changed. This aspect can however, be exactly accounted for analytically: we can determine the amount of substrate species in the intervening space. The amount of species in the intervening space is equal to the average of edge concentrations of the species (right edge of first compartment and left edge of the second), multiplied by the length of the intervening space. This is determined analytically (see Supplementary Information). Now, in the thin compartment regime, the edge concentrations of species in a compartment are practically equal to the average concentration of the species in the compartment. This means that we can account for sequestration through an analytical equation, which depends only on compartmental concentrations. This very substantially improves the predictive capability of the compartmental model (Fig. 1(d)). Furthermore, since the conservation condition is explicit in terms of model variables, this modified compartmental model can easily be used for sensitivity and bifurcation analysis. Note that the factor which prevented an exact match of the compartment model with the PDE model in the non-mass action kinetic regime (errors introduced in averages through nonlinearity), becomes insignificant in the case of thin compartments, and so we expect this augmentation to be effective in both mass-action and non-mass action cases.

#### Non-thin compartment: A new factor emerges

**Figure 2:**
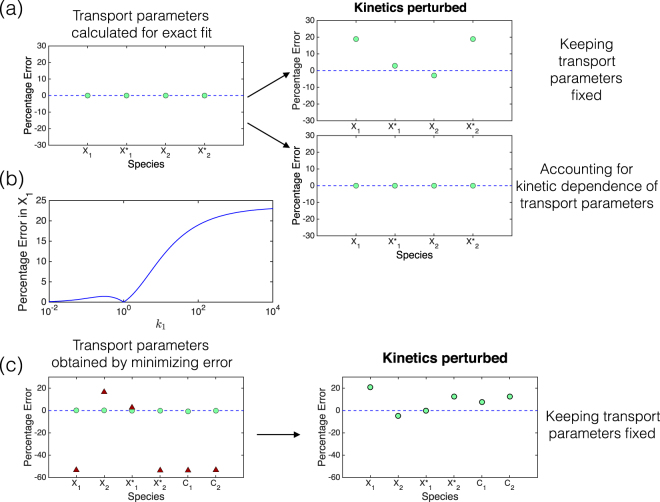
The transport parameters that produce an exact match with the PDE depend on kinetic parameters (non-thin compartment regime). **(a)** In the mass action regime, transport parameters can be computed, which can guarantee an exact match, provided that sequestration in the intermediate space is exactly accounted for. However, this exact match is not robust to changes in kinetics, even if sequestration is correctly accounted for. On the other hand, if we account for the kinetic dependence of the transport parameters, the compartmental model continues to produce an exact fit. **(b)** The percentage error in *X*_1_ (average concentration of *X* in compartment 1) is zero at the basal value of kinetic parameter *k*_1_, and increases when *k*_1_ is perturbed. **(c)** Away from the thin compartment regime, *D*/*LL*_1_ is a poor choice of transport parameter (triangles). In the non- mass action regime, an exact fit is impossible, but transport parameters may be computed by minimizing the error (sum-squared error). Transport parameters obtained by minimizing the error produce a good match (green circles - maximum error 0.6 percent). However, for these transport parameters, the ability to match the PDE is lost if the kinetics is perturbed.

In this case we note that the transport parameter associated with a species is not *D*/*LL*_1_. As we have seen above, there is an exact match (mass-action kinetics) and close to exact match (non-mass action kinetics) for basal kinetic parameter values (having calibrated the model by accounting for sequestration in the intermediate space under basal conditions). When the compartmental model is used in a predictive way through variation of kinetic parameters there is a steadily increasing error (see Fig. 2(a,b)). Sequestration does contribute to this, as seen above. If we adopt the approach above to systematically account for sequestration, we find that, while the amount in the intervening space can be written in terms of compartmental edge concentrations of species, this cannot in general be written exactly in terms of average compartmental concentration. Therefore, there will be a level of approximation associated with this approach to accounting for sequestration.

If we examine a special case where the intervening space is much smaller than the compartmental size, we expect the effect of this sequestration to be minimized. Interestingly even here, the model shows kinetic non-robustness, with a steadily increasing error as kinetic parameters are changed (this is true even when the kinetics is mass-action). Thus, there has to be another contributing factor. This factor is in fact, the transport parameter.

#### What the transport parameter represents

**Figure 3:**
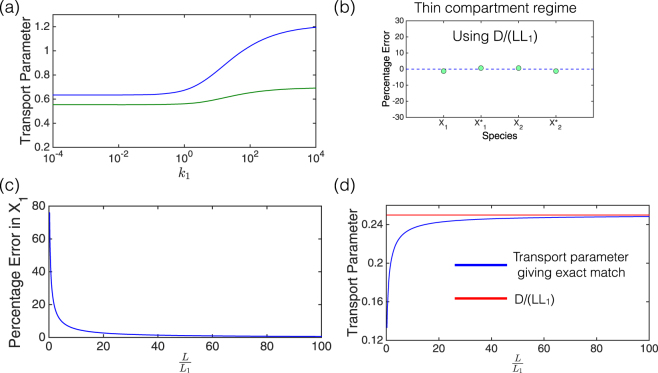
Kinetic dependence of the transport parameters in the mass action regime. **(a)** The transport parameters that produce an exact match between the compartmental model and the PDE, depend on kinetics (see text). For a given change in kinetics, the corresponding variation of transport parameters depends on compartment sizes relative to the intermediate space- green line: $\frac{L}{{L}_{1}}=1$; blue line: $\frac{L}{{L}_{1}}=0.1$. **(b)** Using *D*/*LL*_1_ as the transport parameter produces a good approximation in the thin compartment regime (the maximum error here is about 1 percent). **(c)** Using *D*/*LL*_1_ as the transport parameter, away from the thin compartment regime (small *L*/*L*_1_), can result in a significant mismatch between the compartmental model and the PDE model at steady state. **(d)** The effect of varying $L$ and consequently *L*/*L*_1_, keeping *D*/*L* constant. Away from the thin compartment regime (small *L*/*L*_1_), the transport parameter that produces an exact match differs significantly from *D*/*LL*_1_. It approaches this value as *L*/*L*_1_ becomes large (i.e. as the compartment sizes become smaller relative to the intermediate space).

Examining the PDE model in the case of mass action kinetics, and averaging over compartments, indicates that compartmental average concentrations at steady state do satisfy a compartmental-type equation (see Supplementary Information). In other words, the actual average concentrations in the PDE can emerge as the steady state of the compartmental ODE model. This establishes a correspondence between the PDE model and the compartmental ODE model. What emerges from this correspondence, however, is the fact that in such a case, the transport parameter in the corresponding ODE model demonstrates a clear dependence on both diffusivity and kinetic parameters, but is independent of the total amount of substrate. This means that when kinetic parameters change, so do the transport parameters. In fact, we determine transport parameters which are applicable across the spectrum of compartmental sizes and reduce to *D*/*LL*_1_ in the thin compartment case (see Supplementary Information). Incorporating the kinetic dependence of the transport parameter, results in a substantially reduced error (which would be zero if sequestration was exactly accounted for). Additionally, if the intervening space is small, the effect of distortion due to sequestration is also minimized, and in this case, the compartmental model with kinetically-dependent transport parameters provides a good approximation, and the error is close to zero. It is worth pointing out that the transport parameters of different species, determined as above, are in the ratio of their diffusivities, even though the transport parameter is not proportional to the diffusion coefficient. We also note that the transport parameter is in general not directly proportional to 1/*L* either. The variation of the transport parameter with kinetics, and the error incurred in using a transport parameter *D*/*LL*_1_ is depicted in Fig. 3(a) and (c).

In the non-mass action kinetics regime, while the correspondence of the PDE with a compartmental ODE is not exact, the transport parameters that produce the closest match show a clear dependence on kinetics, echoing the mass-action case. There are two ways to account for this kinetic dependence: one is to do this computationally. This then makes the compartmental model solution as involved as solving the PDE. The other option, which is approximate, is to use transport parameters and their dependence on kinetic parameters, from the mass action regime.

Taken together, by making a correspondence between the PDE model and the compartmental ODE model, we find that in the mass action kinetics case, the average compartmental concentrations of species in the PDE emerge exactly as the steady state of the compartmental ODE model, provided that (i) the transport parameters have a particular dependence on kinetics (and diffusivities and sizes), which can be determined, (ii) the effect of sequestration is exactly accounted for.

#### Open reaction systems

**Figure 4:**
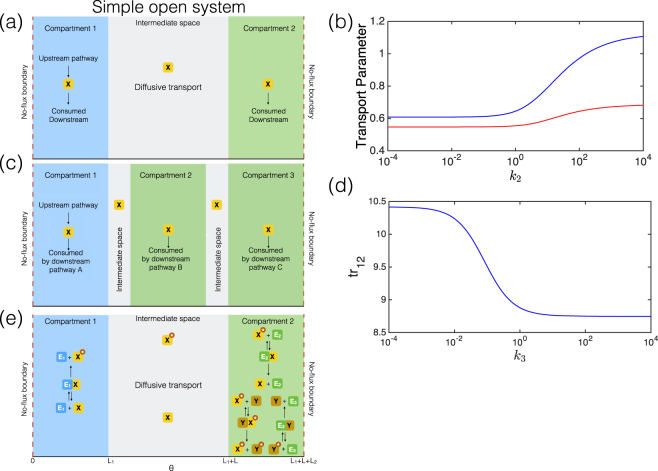
Simple open system, and pathways with added complexity. **(a)** Schematic for a simple open system with production in the first compartment and degradation in both first and second compartments. **(b)** For a given change in kinetics, the corresponding variation of transport parameters, that gives an exact match, depends on compartment sizes relative to the intermediate space- red line: $\frac{L}{{L}_{1}}=1$; blue line: $\frac{L}{{L}_{1}}=0.1$. **(c)** Open system with *X* produced in compartment 1 and consumed in compartments 1, 2, and 3. **(d)** Using the analytical solution to the PDE model, we see that modulating the kinetics in compartment 3, can alter the transport parameter (that gives an exact match) associated with transport of *X* between compartments 1 and 2 (tr_12_). **(e)** Schematic for the comparmentalized two-step cascade.

For completeness, we also consider a sample open reaction pathway, with production of a substrate (“inflow”) in one compartment, and degradation “outflow” in both the first and second compartments (Fig. 4(a,b)). In this case, there is no conservation condition, and so sequestration does not play a role. Exactly as seen above, in the thin compartment case, the transport parameter of a species is *D*/*LL*_1_. In the non-thin compartment case, for mass action kinetics (of degradation) the transport parameter can be determined as a function of kinetic parameters (though it is independent of the rate of production), diffusivity, and sizes of the compartments and the intervening space. This can be done analytically, and is discussed in the Supplementary Information. Therefore in this case, the model using the modified transport parameters can be used to obtain an exact match.

#### Comments on sequestration

A number of compartmental models employed in the literature, including ones which implicitly work in the thin compartment limit (using a transport parameter *D*/*LL*_1_), do not systematically account for sequestration. In fact the effects of sequestration are strongest in the thin compartment regime. Our analysis above shows how this can be accounted for at steady state analytically, while still keeping the focus on the reaction compartments. It is possible to introduce the intervening space as another compartment, and arrive at the same result, through this expanded model (the equivalence of the two approaches is demonstrated in the Supplementary Information). We note, in passing, that in certain special cases (Mass action kinetics, equal diffusivities), the total amount of species in the compartments can be determined directly (see Supplementary Information). One aspect common to both the 1-D case and higher dimensions is the fact that focussing only on the reaction compartments (rather than introducing the intervening space as another compartment) allows for greater conceptual simplicity, while accounting for sequestration. Note that having additional compartments requires the computation of additional inter-compartmental transport parameters in all these cases. Incidentally, it is worth pointing out that in 2-D, when the number of reaction compartments is greater than 2, introducing the intervening space as another compartment may lead to a simplification of the model structure.

#### Comments on the transport parameter

Our analysis in the mass-action kinetic regime demonstrates a steady state correspondence between the PDE model and a compartmental model with transport parameters having kinetic dependence (which can be determined). This has multiple important consequences. Errors introduced by using a compartmental ODE model with fixed transport parameters can be seen by comparing it with another ODE model of the same type, thus explicitly revealing the source of the disparity. The mass-action regime allows us to explicitly lay bare a factor at play, which is equally relevant in the non-mass action regime. The factor responsible for the disparity is the fundamental reason for the degradation in predictive capacity of such a model when kinetic parameters are changed, and is revealed explicitly through our analysis. Another qualitative effect explicitly revealed by the kinetic dependence of transport parameters in the “corresponding” ODE model, is discussed in the next section.

#### Relevance to Systems and Synthetic Biology

We note that the biochemical modules studied above are basic building blocks of complex pathways in systems biology. Sequestration in particular has been a recurring theme in the context of signalling, in the past decade^33,34^. The fact that the transport parameter (which may guarantee an exact match) is not constant (even with mass action kinetics) indicates a fundamental trade-off in modelling: choosing a simpler framework necessarily needs non-constant transport parameters to guarantee accuracy, away from the thin compartment regime. Further, in both systems and synthetic biology there is an interest in the effect of multiple “levers” in affecting pathways. Thus it is clear that having a description which is not robust to changes in kinetic parameters is fundamentally limiting. Finally, depending on the context, either the regime of thin compartments (eg: poles of bacteria) or the regime of relatively large compartments separated by small intermediate spaces (calcium microdomains^13^, multi-compartment vesicles) may be encountered.

### Extensions to more complex systems

Having studied the behaviour of compartmental ODE models of simple open and closed systems, we expand our study to a range of more complex scenarios with a view towards building a bridge towards applications in systems and synthetic biology. To that end we study (i) A two tier enzymatic modification cascade, which is an extension of a covalent modification cycle (ii) The effect of introducing additional compartments, as an example of a modular strategy of spatial organization in synthetic biology (iii) Multisite substrate modification, which is another distinct extension of covalent modification cycles (iv) A pathway design problem relevant to both metabolic engineering and synthetic biology. Each of these scenarios presents a new context/application, while also revealing new consequences of the effects studied in the previous section.

#### A two tier cascade

We now build on the above results to examine slightly more complex reaction systems. Thus far we have considered only a single tier of a modification cascade. We now briefly examine what happens when we have multiple stages of modification. To do this, we consider a two stage closed system, where the first modification tier occurs through mass action kinetics, and the second tier is completely localized in the second compartment (Fig. 4(e)). The modified substrate of the first tier, serves as an enzyme (kinase) for the second tier. This is representative of biological settings where downstream pathways are activated by the modified substrate at the first stage. It is already known that a particular modification stage can affect a stage above it, an effect referred to as retroactivity. This in turn, arises because some of the substrate species in the first tier is sequestered in a complex downstream. As before we look at two regimes.

#### Thin compartment

In this case, the transport parameters are *D*/*LL*_1_ (the same analytical calculation holds good), and the additional sequestration of the first tier output in the second tier of the cascade is accounted for by describing the second tier modification in the compartmental model. The essential insight is that profiles of all species within a given compartment are practically flat. This means that (i) sequestration in the intermediate space can be easily accounted for (ii) the nonlinearity associated with the sequestration downstream leads to negligible error. Therefore, the compartmental model can achieve a close match to the PDE model with regard to the diffusing species, and consequently also for the output of the cascade.

#### Non-thin compartment

Here, as noted earlier, the transport parameters for the diffusing species are no longer *D*/*LL*_1_. Looking at the first stage of the modification, we see that the only change introduced by the second tier modification, is to reduce the total amount of substrate species in the first step, due to an additional sequestration. Now, as discussed above, the transport parameters (i.e transport parameters in the compartmental ODE model which would allow for an exact match with the PDE model) of the diffusing species in the first stage of the modification depend on diffusivity, compartment sizes, size of intervening space, but not the total amount of species. Thus the transport parameter remains exactly the same. In other words, through this choice of transport parameters, and exactly accounting for sequestration of the species of the first tier, both in the intervening space and downstream, one can ensure that the compartmental ODE model exactly matched the PDE model in the first tier. In this case, there is however, a certain degree of error in analytically trying to account for the sequestration, both in the intermediate space (since we cannot approximate edge concentrations by averages) and in the second tier (due to the nonlinear kinetics). Both of these can be traced to the fact that concentration profiles are not uniform in a compartment.

The essential insight from the above is that the effect of adding a second tier of modification localized in one of the compartments has two consequences: (i) The transport parameters of the diffusing species (which move between compartments) in the“corresponding” compartmental ODE model, is unaffected by the second tier reaction. This is relevant in many biological contexts, where the output of one stage may be involved in triggering many pathways downstream (ii) There is an effect of sequestering of the output of the first tier downstream. As long as the downstream reaction is not in the mass action regime, the second tier reaction introduces a nonlinearity which prevents an exact match between the compartmental model and the PDE.

#### A modular approach to compartmentalization

In trying to design metabolic pathways, it is natural to attempt a modular design through spatial organization. We examine such a case here, building on the simple open system examined previously. We noted that it was possible to obtain a compartmental description of this system, even when the compartments were not thin. We now consider an augmentation of the original system, with an extra compartment downstream (where the substrate degrades). Analyzing this system exactly as before shows that it is possible to obtain a compartmental description similar to that above. Strikingly, here the transport parameter (which guarantees an exact match with the PDE) associated with transport between the first and second compartments is actually altered by the kinetics in the third compartment (Fig. 4(d)). This illustrates that adding a compartment downstream can actually affect transport parameters upstream, even though it does not really affect the transport upstream. This further highlights how the transport parameter in general depends on both kinetics and diffusivity, and demonstrates that global changes in kinetics can effect local transport parameters.

The essential insight here is that since transport parameters (i.e. those which can guarantee exact matches with the PDE) in general depend on kinetics (of all reactions), a change in the kinetics in the third compartment can affect the transport parameters associated with the first and second compartments. This follows from our study of the previous section. This occurs generally as long as (i) the system is not in the thin compartment regime (transport parameters would be independent of kinetics) and (ii) the diffusivities are not large for the length scale of the system (otherwise spatial gradients would not be sustained). Both conditions can occur in a wide range of contexts.

#### Multisite modification: Transport parameters and diffusivity

**Figure 5:**
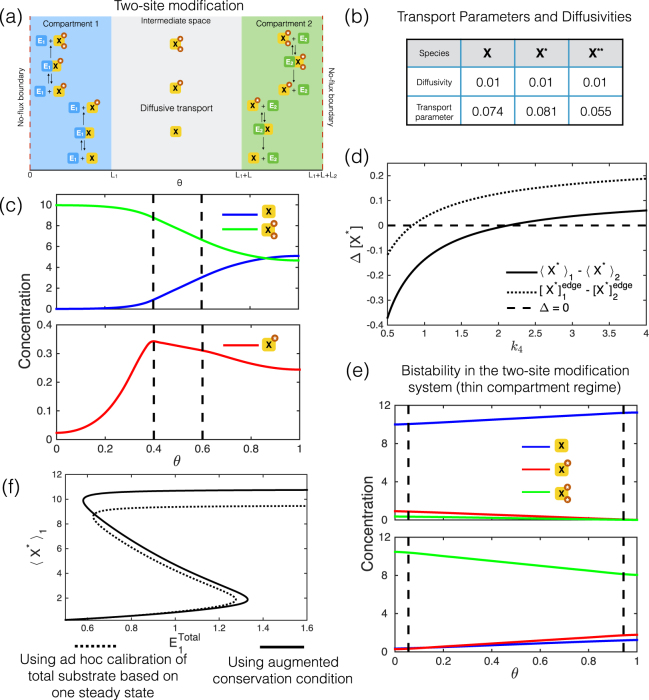
Multi-site modification. **(a)** Schematic for compartmentalized distributive two-site modification pathway (kinase in compartment 1 and phosphatase in compartment 2). **(b)** With multiple diffusing species in a pathway (two-site substrate modification: see text), their associated transport parameters, that produce an exact match, need not be in the same ratio as their diffusivities, even when the species belong to the same conserved pool. In **(c)** and **(e)**, where steady state concentration profiles are shown, the dashed lines mark the compartment boundaries. **(c)** An example of steady state concentration profiles where [X*] is clearly non-monotonic, and the difference in edge concentrations and the difference in average concentrations between compartments, have opposite signs. Note that the maximum of [X*] is inside compartment 1 and not on the boundary. **(d)** Plot showing how the difference in average concentrations and the difference in edge concentrations vary with kinetics (rate constant *k*_4_). Notice that they have opposite signs over a certain range of *k*_4_. **(e)** The two-site modification pathway shows bistability with compartmentalized enzymes (The system considered is in the thin compartment regime: $\frac{L}{{L}_{1}}=18$). Spatial concentration profiles of the modified forms, for the two different stable steady states are shown. **(f)** Comparing a compartmental description that systematically accounts for sequestration in the intermediate space with another which is calibrated to account for total amount of species in the compartments in an *ad hoc* manner (the calibration is based on one of the two steady states at basal conditions: ${E}_{1}^{Total}$ = 0.6). We find that the model with *ad hoc* calibration introduces significant errors in capturing the other steady state. In fact, under basal conditions, the model with *ad hoc* calibration does not even exhibit a second steady state.

We now examine a second type of complexity in the reaction pathway: multisite phosphorylation of substrate by a kinase-phosphatase pair. In contrast to the simple covalent modification cycle considered above, we have two possible modifications, and consequently three substrate species which cycle between compartments (Fig. 5(a)). This represents a different kind of extension of a covalent modification cycle: one where there are additional cycles of substrate modification. Analyzing this system (and obtaining a correspondence between the PDE model and compartmental ODE models) shows that we can obtain a compartmental model which exactly matches the PDE at steady state, when modifications are through mass action kinetics. The transport parameters, in such a case, in general can depend on the kinetic parameters, but interestingly, the transport parameters of different species are not in the ratio of their diffusivities. For instance, as shown in the table in Fig. 5(b), equally diffusing species have different transport parameters. This insight, which stems from the influence of kinetics on transport parameters, is valid for other reaction systems as well.

#### Compartmental averages and the direction of transport

Our analysis of the multisite phosphorylation pathway above reveals a new feature hitherto not encountered. Even when the kinetics is in the mass-action regime (and even when diffusivities of species are equal), we find (see Fig. 5(c)) that non-monotonic concentration profiles are possible within a compartment. This has the important consequence that there can be a significant difference between compartmental edge and average concentrations. Furthermore, the difference between (nearest) edge concentrations of two compartments may not even have the same sign as the difference in average concentrations (Fig. 5(d)). Diffusive transport in the intervening space is driven by the difference in edge concentrations, while transport is conceptualized in the compartmental model in terms of differences in average concentrations. This has the consequence that in such situations, the transport parameter (to exactly fit the PDE solution) has got to be negative. In other words, populating a compartmental model with positive transport parameters will be guaranteed to lead to a mismatch. This is because it is fundamentally incapable of capturing both average concentrations and the direction of transport simultaneously, for certain species. Building on this, we find that it is possible for (a) nearest edge concentrations to be the same, while average concentrations are different, in which case the transport parameter is zero (b) nearest edge concentrations are different while the average concentrations are the same, in which the transport parameter (which guarantees a match with the PDE) diverges. Taken together this shows that there may be fundamental limitations to compartmental ODE models exactly matching the PDE model, if the transport parameters are positive. We note in passing, that having transport parameters being negative could lead to unphysical behaviour such as negative concentrations, in dynamic simulations.

#### Multistability in compartmentalized biochemical pathways

Multisite phosphorylation systems have attracted a lot of attention, because of their potential to exhibit complex information processing characteristics, such as bistability^35,36^. When we consider multisite phosphorylation in thin compartments, in a kinetic parameter regime which allows for bistability, we readily find two different steady states (Fig. 5(e)). Note that these two steady states do not have the same total amount of substrate in the two compartments. This means that any ad hoc, or even experimentally based calibration of the compartmental model to account for sequestration, will necessarily fall short in capturing the two steady states (even in a relatively thin compartment) (Fig. 5(f)). On the other hand, in this case, using the analytically augmented sequestration condition, allows for both steady states to be accurately captured.

Another aspect of bistability is worth highlighting in the context of compartmental systems. In our earlier study (single covalent modification, non-mass action kinetics, non-thin compartment) we noted that a compartmental model would not exactly match a PDE model. We demonstrated that we could obtain transport parameters using an optimization approach, to realize a “best fit”. When there are potentially multiple steady states, getting the transport parameters to best fit a given steady state in the compartmental model with that in the PDE may still allow for the possibility of other steady states (though this is not guaranteed). However with this choice of transport parameters, the second steady state of the compartmental model may not optimally match that of the PDE (or even be close). This means that if a single set of transport parameters is used with a desire to accurately obtain both steady states, then the best fit criteria must necessarily simultaneously incorporate both steady states.

The essential insights from our study of the multisite substrate modification are as follows. (i) With two species diffusing (i.e a single covalent modification cycle, mass action regime, studied earlier), the transport parameters of the “corresponding” ODE model are in the ratio of their diffusivities. The number of species in this case places a mathematical constraint which ensures this. When more than two species diffuse (same regime), this constraint is removed, and transport parameters are no longer in the ratio of their diffusivity. (ii) Non-monotonicity in concentration profiles can result in a qualitative mismatch between differences in (nearest) edge concentrations and differences in average concentrations of two compartments. This can occur even in the mass action regime. Consequently a compartmental model with positive transport parameters will result in a mismatch with the PDE. The conceptualization of transport being driven by differences in average concentrations can result in significant errors. (iii) There is considerable interest in the capacity of multi-site modification systems to generate bistability and sequestration plays a central role (even when the system is localized). Consequently, a compartmental model which does not correctly account for sequestration is liable to make incorrect inferences regarding this.

#### Exemplar pathway design problems

**Figure 6:**
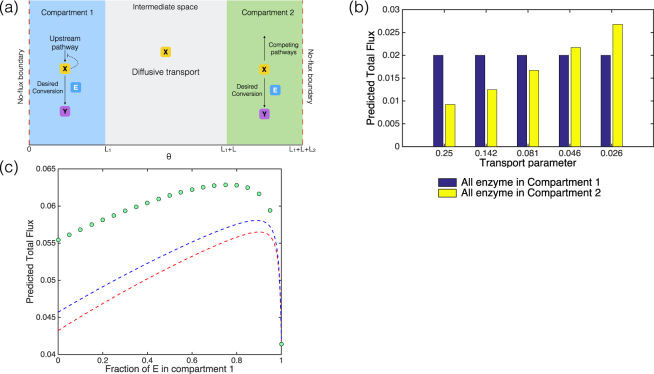
Sample design problem, using compartmental models to optimally target a metabolic enzyme to a compartment. **(a)** Schematic of the compartmentalized metabolic pathway, with upstream pathway in compartment 1 producing a metabolite *X*, and reactions consuming *X* in both compartments, including the desired conversion (by enzyme *E*) and competing pathways. In the text, we examine two scenarios, one with negative feedback inhibition (depicted) and one where this is absent. **(b)** The case of no feedback inhibition. The choice of transport parameter is crucial for the correct prediction of fluxes for the two extreme distributions (no feedback inhibition). An incorrect transport parameter might lead to a completely misleading impression as to which distribution is better. The fluxes are shown as computed using a range of transport parameters between *D*/*LL*_1_ (which is 0.25 here) and the transport parameter accounting for the basal reactions (which is 0.026). **(c)** The case with feedback inhibition. There is significant disparity between the flux of product obtained from the compartmental model and the PDE. The green dots represent the flux of the desired reaction, as given by the PDE. If a transport parameter *D*/*LL*_1_ is used in the compartmental model, the disparity is substantial (red dashed line), and further, even an improved estimate of the transport parameter (accounting for the basal reactions) still leaves a clear degree of disparity (blue dashed line).

A particular interest in compartmentalization comes from metabolic engineering, where compartmentalization (for example manipulating or reorganizing pathways within naturally available compartments) offers a potentially important tool for manipulating metabolic systems^37^. We examine two exemplar cases related to this. In the first case, a metabolite is produced in one compartment, diffuses to the other and is consumed in the second compartment (an undesired pathway). An enzyme (of fixed amount) is localized in one of the compartments resulting in the conversion of the metabolite to a desired product there. The goal is to determine, which compartment the enzyme *E* should be targeted to, to maximize the flux of the desired conversion. Questions of this kind have been examined in multiple instances in the literature^38^. As seen in Fig. 6(b), a choice of transport parameter *D*/*LL*_1_ results in a conclusion which is opposite to that suggested by the PDE model. On the other hand, incorporating the effects of the basal production/transport/consumption in the transport parameters of the compartmental model, results in the right conclusion. This shows how a plausible but incorrect choice of transport parameters can qualitatively affect the result. We note that the second reaction (desired pathway mediated by the enzyme) will affect the transport parameters as well. For the greatest accuracy, it may be necessary to incorporate the effects of this pathway in the transport parameter.

In the second exemplar case, we consider the setup above, but with an extra ingredient: the substrate inhibits its production (Fig. 6(a)). This is observed in multiple contexts. Here we examine what the optimal distribution of enzyme between the compartments should be, to maximize total flux of the output. Here as seen in Fig. 6(c), we find that there is a significant disparity between the flux of product obtained from the compartmental model and the PDE. Furthermore, the compartmental model predicts that if all the enzyme is in either of the compartments, the flux is roughly the same, a conclusion which is misleading. We note that if a transport parameter of *D*/*LL*_1_ is used in the compartmental model, the disparity is substantial, and further, even an improved estimate of the transport parameter (as done above) still leaves a clear degree of disparity. In such cases, it is necessary to incorporate the effects of the second reaction on the transport parameter to obtain reliable predictions.

The essential insight is that non-trivial pathway optimization problems usually involve a trade-off between multiple competing factors. When the compartments are not small (i.e. transport parameters are not fixed) and when the diffusivities are not large (i.e. non-trivial gradients can exist), the kinetic dependence of transport parameters (which guarantee a fit with the PDE model) can significantly affect the balance between competing factors in determining the optimal solution. In such a case the optimal solution may be very different from that in a case where the transport parameters were constant. Consequently there can be an essential mismatch between the optimal solution in the PDE model and that of a compartmental ODE model with constant transport parameters.

#### Relevance to Systems and Synthetic Biology

Even in more complex synthetic and systems biology applications, the presence of multiple levers enhances the applicability of the associated pathway, and their role needs to be accurately understood. Optimizing and manipulating pathways is a major theme in synthetic biology and metabolic pathways, and compartmentalization is seen as a vital tool. Compartmentalization of modules is seen as a basic ingredient in the realization of proto-cells. In all these cases, the choice of a modelling framework must enable accurate understanding and design. Finally, design (whether of multiple compartments or multiple pathways) is usually implemented in a modular way, and our analysis indicates the pitfalls associated with that, in the context of compartmentalization.

## Discussion

Compartmentalization is a basic ingredient of cellular systems and a key focal point of interest, for multiple reasons. This stems from the role of compartmentalization in cellular information processing networks, compartmentalization as a key ingredient in evolution, and the interest in using compartmentalization as a key engineering “lever” to regulate and design biochemical pathways. It is clear in all these cases, that progress needs both dedicated experiments as well as modelling and systems approaches. Compartmental ODEs are a popular choice for models, as they incorporate essential features about compartments, focus on the reaction compartments (which are the focus of interest anyway) and are tractable: they represent a manageable balance between complexity and analyzability. However since the modelling framework sits right at the foundation of the investigation and serves as the basis for analysing and designing compartments, it is vital that effect of the choice of modelling framework in affecting the results is well understood.

We analyzed compartmental ODE models compared with detailed PDE models in a range of systems, with reactions in two compartments, separated by an intervening space. We spanned an entire range, with thin compartments (with substantial intervening space) at one extreme, and fat compartments (with relatively small intervening space) at the other. We primarily compared the models at steady state. Our main conclusion is that there are two factors which can significantly affect whether compartmental ODE models yield accurate results: correct determination of total compartmental concentrations (something which is independent of a model) and the transport parameters (which arise from the model description). In the thin compartment regime, due to conservation of species, there may be a considerable amount of species in the intervening space, and further this amount may depend on reaction kinetics and other parameters. We demonstrate how analytically incorporating the conservation results in an augmented compartmental model, which will give much more accurate results. In the other regime, one of the key factors which affects the accuracy of the compartmental models is the choice of transport parameters. Through a careful analysis of the correspondence between compartmental ODE models and PDE models (with mass action kinetics), we find that the the steady state solution of the PDE model corresponds to that of a compartmental ODE model where the transport parameters actually involve a combination of diffusion and kinetics. Consequently any compartmental model in this regime, which incorporates constant transport parameters will make predictions which are not robust to changes in the kinetics. For compartmental sizes which are at neither extreme, both factors can contribute significantly. We note in passing, that reductions from PDE to ODE models have also been considered in other settings, such as fronts in reaction-diffusion systems, in the context of cell polarization^39,40^.

These same core factors play very significant roles when biochemical pathways with additional complexity are considered, whether multisite substrate modification, the effects of bistability, the effects of adding further compartments, or designing metabolic pathways to maximize yield. In general, when the kinetics of the reactions follow mass action kinetics, it is possible to obtain a compartmental model, with transport parameters which depend on kinetics, to exactly match the PDE model at steady state. As seen from our studies, in certain situations even with mass action kinetics, non-monotonic concentration profiles may arise in compartments, and consequently the gradient of species between compartments may not be in the direction from higher average concentration to lower average concentration. In other words, there may be a fundamental limitation in such cases for a compartmental ODE model with positive transport parameters, to match the PDE model and this can be traced to a mismatch between how the “driving force” of transport is conceptualized in compartmental ODE models and how it actually occurs. When the kinetics is strongly nonlinear, this factor can prevent an exact match, for fundamental reasons. We note that sequestration is a dominant underlying theme in theoretical and experimental studies of signalling in the past decade. From our results, we note that for compartmental models to make accurate predictions and conclusions, both these factors must be appropriately incorporated, failing which very significant inaccuracies could result.

Our approach has involved systematically dissecting and analyzing compartmental systems proceeding from the simple to the complex, while also studying both closed and open systems, kinetic regimes, compartment sizes, as well as a range of more complex scenarios. Through this unified approach, we are able to systematically and explicitly disentangle multiple contributing factors, to better understand their interplay. As a consequence, we are (i) able to identify some very basic sources which limit the efficacy of compartmental ODE models (ii) identify how and when that can be corrected for (iii) obtain clear insights relevant to each category of model/context (iv) sharply reveal what the consequences of additional complexities may be in this regard, and how they depend on the type of augmentation.

Our focus in this paper was on the steady state behaviour of compartmental models. It is clear that when dynamic behaviour of models is concerned, there will in general be additional differences between the compartmental model and the PDE model (so that even if steady state behaviour is the same, the dynamic behaviour may be different) This in turn is due to the essentially different way in which the transport is described. Naturally, if there is a mismatch in steady state behaviour, there will have to be a difference in dynamic behaviour as well. We point out that if dynamic behaviour is desired, it would be preferable to describe the intermediate space as an additional compartment.

Cellular systems are complex and modelling in systems biology necessarily involves simplifications: what is the relevance of our analysis here? Compartmental models of biochemical pathways are already being used in many contexts simply because it is recognized that compartmentalization is a vital ingredient, playing a potentially important role. This being the case, a compartmental ODE representation runs the risk of severe qualitative and quantitative distortions. Sequestration and conservation have already been shown to be a key ingredient in qualitatively altering signal transduction characteristics in multiple contexts^33,36^. The fact that the transport parameter (guaranteeing an exact match) in such a representation (when the compartments are not thin) actually depends on kinetics, has serious and profound implications. It simply indicates, that at this level of description (to ensure an exact match) the inter-compartmental transport parameters and reactions are not independent, and multiple entities in a reaction network can affect the transport parameters. In closed systems, there is, in fact, no regime of compartmental sizes, which is immune to both these distortions. In synthetic biology, engineering compartmentalized pathways necessarily progresses starting from simpler tractable cases. Given the centrality of compartmentalization in the design goal and the relative simplicity (in the initial stages of the setup), adopting a naive compartmental ODE model is harder to justify.

We now examine a range of contexts of current interest in compartmentalization, which goes beyond understanding their effect in cellular information processing. In cell-free synthetic biology, compartmentalized biochemical pathways, including coupled systems of lipid vesicles and spatially coupled compartments on silicon chips, have been engineered. Cell-free transcription-translation systems encapsulated in lipid vesicles^41^ and compartmentalized biochemical modules^42^, have been investigated in the context of building artificial cells. Spatially organized chemical systems, relying on compartmentalization and diffusive transport, are a central theme in exploring the origins of life. Active droplets, with chemical reactions compartmentalized by phase separation, and maintained far from thermodynamic equilibrium by the supply of a chemical fuel, have been shown to be capable of spontaneous division, and suggested as candidates for early proto-cells^43^. Optimizing spatial organization of metabolism is an emerging theme in synthetic biology^44^. Among the natural strategies used by a cell are spatial organization and compartmentalization at multiple levels - pathways compartmentalized at the intercellular level (different pathways in different cells e.g. symbiotic microbial communities), reactions compartmentalized at the level of sub-cellular compartments (peroxisomes, mitochondria in eukaryotes; proteinaceous microcompartments in prokaryotes), and enzymes localised at the level of multifunctional enzyme complexes by scaffold proteins. These strategies have been the basis for synthetic biology designs including engineering microbial co-cultures^45^ -where the focus is on realizing complex processes and achieving new functions without genetic engineering by spatially organizing communities of multiple microbes. Designing the spatial ordering of metabolic pathways in communities of competing/cooperating microbial cells, cross-feeding between co-operating strains of *E. coli* ^46^ and engineered cross-feeding of essential metabolites between yeast strains resulting in a synthetic cooperative system within a yeast population are different directions which are being pursued. Finally, another tool in metabolic engineering is manipulating the natural compartmentalization of pathways^47–50^.

By comparing and establishing a correspondence between compartmental ODE and PDE models for various classes of typical reaction systems, we (i) provide analysis which can be employed in using these models, (ii) establish limits and cases where the compartmental ODE models do and do not work, (iii) present augmentation which reduces/eliminates the mismatch between the ODE model and the PDE, (iv) suggest better procedures for the use of these compartmental models. We discuss this last point briefly. In general, in a system of diffusive transport, directly using a PDE model, along with its tools of analysis, may be preferable. If the advantage of a compartmental model is desired, then another possibility is that it can be used (with potential augmentations) alongside a PDE model, so that the relative advantage of each approach can be employed. For some applications, the situation may be such that only a compartmental model may be feasible. In such a case our analysis may still suggest ways in which it can be used more effectively. For instance (say in a non-mass action regime) the question arises as to how to choose the transport parameter: our analysis (for non-thin compartments) indicates that the transport parameter would depend on kinetics. In such a case it may be possible to obtain “best estimates” of transport parameters at different points over a range of kinetic parameters, and then interpolate (rather than just use one basal value). In the context of pathway optimization, it may be possible to at least do some *a posteriori* analysis of the robustness of results to changes in transport parameters. Depending on whether the resulting behaviour is robust or not, the modelling approach may need to be refined or revisited. Finally, even if the focus is on compartmental behaviour, obtaining data which resolves the spatial behaviour in a compartment, may be a useful way of performing *a posteriori* checks.

Any modelling/systems framework involves a trade-off between tractability and effective representation of important features. A compartmental ODE model is a tractable framework which aims to capture the essential features of compartmentalization reliably. Our analysis indicates that while a compartmental ODE model may be useful as a nominal description in systems biology, one must be very careful about when it can and cannot be applied, and also when it can be used in a predictive manner reliably. In multiple instances, even with all the calibration, data fitting and parameter estimation this model will fall short for fundamental reasons. In some cases it can be augmented or improved, and our analysis indicates exactly how that can be done. In the context of engineering compartments, our study indicates when this type of model is and isn’t a reliable framework for undertaking any systematic design. In the ultimate analysis, getting the interaction between the physics and chemistry involved in compartmentalization right, should be the key to successfully understanding and engineering compartmentalization, and this should dictate the choice of the foundational framework which is the basis of all the investigations.

### Data availability

All data generated or analysed during this study are included in this published article (and its Supplementary Information files).

## Electronic supplementary material

Supplementary Information 
