# Supplementary material for: Modelling compartmentalization towards elucidation and engineering of spatial organization in biochemical pathways
Source: Sci Rep. 2017 Sep 21;7:12057. doi: 10.1038/s41598-017-11081-8 (PMC5608717; doi:10.1038/s41598-017-11081-8)
Supplement: Supplementary file 1 — Supplementary Information [file 41598_2017_11081_MOESM1_ESM.pdf]

Modelling compartmentalization towards elucidation and engineering of spatial organization in biochemical pathways

Govind Menon [1], Chinedu Okeke [1] and J. Krishnan [1,2].

[1] Chemical Engineering and Chemical Technology, Centre for Process Systems Engineering and
[2] Institute for Systems and Synthetic Biology, Imperial College London, South Kensington Campus, London SW7 2AZ, UK.

Supplementary Information

The supplementary information is organized as follows. We first outline the models for the compartmentalized pathways that we examine. Following this, we present a detailed analytical investigation of multiple aspects discussed in the main text. We also present additional information related to the plots shown, including the parameter values used. We note that the analytical solutions in all relevant cases have been cross-validated against numerical solutions of the PDE models.

1 Models

The different biochemical pathways examined here are described below (for the two compartment scenario). Schematics for the various pathways are presented in the main text and Figure S1.

1.1 Single modification cycle

This is a closed system consisting of a single enzymatic modification cycle involving species X and X^*. Both species diffuse across the whole domain, and may have different diffusivites. E_1, the enzyme catalyzing $X \to X^*$ is non-diffusing and uniformly distributed in compartment 1 (see Figure 1(a)). E_2, the enzyme catalyzing $X^* \to X$ is non-diffusing and uniformly distributed in compartment 2. The enzyme substrate complexes, XE_1 and X^*E_2 (referred to as C_1 and C_2) are also non-diffusing. The total amount of substrate within the whole domain, consisting of free substrate (X and X^*) and the complexes, is conserved.

PDE model: In compartment 1 ($0 \le \theta \le L_1$),

$$\begin{aligned}
\frac{\partial[X]}{\partial t} &= -k_1[X][E_1] + k_{-1}[C_1] + D_X \frac{\partial^2[X]}{\partial\theta^2} \\
\frac{\partial[X^*]}{\partial t} &= k_2[C_1] + D_{X^*} \frac{\partial^2[X^*]}{\partial\theta^2} \\
\frac{\partial[C_1]}{\partial t} &= k_1[X][E_1] - k_{-1}[C_1] - k_2[C_1]
\end{aligned}$$

Between the compartments ($L_1 \le \theta \le L_1 + L$),

$$\begin{aligned}
\frac{\partial[X]}{\partial t} &= D_X \frac{\partial^2[X]}{\partial\theta^2} \\
\frac{\partial[X^*]}{\partial t} &= D_{X^*} \frac{\partial^2[X^*]}{\partial\theta^2}
\end{aligned}$$

In compartment 2 ($L_1 + L \le \theta \le L_1 + L + L_2 = l$),

$$\begin{aligned}
\frac{\partial[X]}{\partial t} &= k_4[C_2] + D_X \frac{\partial^2[X]}{\partial\theta^2} \\
\frac{\partial[X^*]}{\partial t} &= -k_3[X^*][E_2] + k_{-3}[C_2] + D_{X^*} \frac{\partial^2[X^*]}{\partial\theta^2} \\
\frac{\partial[C_2]}{\partial t} &= k_3[X^*][E_2] - k_{-3}[C_2] - k_4[C_2]
\end{aligned}$$

With the conservation equations,

$$\begin{aligned}
X^{Total} &= (\langle[X]\rangle + \langle[X^*]\rangle)l + \langle[C_1]\rangle_1 L_1 + \langle[C_2]\rangle_2 L_2, \\
E_1^{Total} &= (\langle[E_1]\rangle_1 + \langle[C_1]\rangle_1) L_1 \\
E_2^{Total} &= (\langle[E_2]\rangle_2 + \langle[C_2]\rangle_2) L_2
\end{aligned}$$

where $\langle\cdot\rangle$ represents the spatial average over the whole domain, and $\langle\cdot\rangle_i$ represents the spatial average over the compartment i.

The corresponding compartmental model is as follows.

Compartmental ODE model: In compartment 1,

$$
\begin{aligned}
L_1 \frac{d[X_1]}{dt} &= -k_1[X_1][E_1]L_1 + k_{-1}[C_1]L_1 - tr^{X}_{1\to 2}[X_1]L_1 + tr^{X}_{2\to 1}[X_2]L_2 \\
L_1 \frac{d[X_1^*]}{dt} &= k_2[C_1]L_1 - tr^{X^*}_{1\to 2}[X_1^*]L_1 + tr^{X^*}_{2\to 1}[X_2^*]L_2 \\
\frac{d[C_1]}{dt} &= k_1[X_1][E_1] - k_{-1}[C_1] - k_2[C_1]
\end{aligned}
$$

In compartment 2,

$$
\begin{aligned}
L_2 \frac{d[X_2]}{dt} &= k_4[C_2]L_2 - tr^{X}_{2\to 1}[X_2]L_2 + tr^{X}_{1\to 2}[X_1]L_1 \\
L_2 \frac{d[X_2^*]}{dt} &= -k_3[X_2^*][E_2]L_2 + k_{-3}[C_2] - tr^{X^*}_{2\to 1}[X_2^*]L_2 + tr^{X^*}_{1\to 2}[X_1^*]L_1 \\
\frac{d[C_2]}{dt} &= k_3[X_2^*][E_2] - k_{-3}[C_2] - k_4[C_2]
\end{aligned}
$$

With corresponding conservation equations for the substrate and the enzymes. The form of the conservation equation for the diffusing substrate will be examined as part of our analysis.

In the compartmental ODE equations, the variables $[X_i]$ and $[X_i^*]$ represent the concentrations of species X and X^* in compartment i.

1.2 Two-step cascade

This is a closed system consisting of two enzymatic modification cycles - the first involving species X and X^*, and the second involving Y and Y^* (See Fig. S1(a)). As before, the X cycle involves enzymes E_1 and E_2. Both of these are non-diffusing and uniformly distributed in compartments 1 and 2 respectively. X and X^* diffuse over the whole domain, with X^* acting as the enzyme for $Y \to Y^*$. Y and Y^* are non-diffusing and confined to compartment 2. E_3, the enzyme catalyzing $Y^* \to Y$ is also non-diffusing and uniformly distributed in compartment 2. All enzyme-substrate complexes are non-diffusing. The total amount of substrate over the whole domain, in each of the cycles, is conserved.

PDE model: In compartment 1 ($0 \le \theta \le L_1$),

$$\frac{\partial[X]}{\partial t} = -k_1[X][E_1] + k_{-1}[C_1] + D_X\frac{\partial^2[X]}{\partial\theta^2}$$
$$\frac{\partial[X^*]}{\partial t} = k_2[C_1] + D_{X^*}\frac{\partial^2[X^*]}{\partial\theta^2}$$
$$\frac{\partial[C_1]}{\partial t} = k_1[X][E_1] - k_{-1}[C_1] - k_2[C_1]$$

Between the compartments ($L_1 \le \theta \le L_1 + L$),

$$\frac{\partial[X]}{\partial t} = D_X\frac{\partial^2[X]}{\partial\theta^2}$$
$$\frac{\partial[X^*]}{\partial t} = D_{X^*}\frac{\partial^2[X^*]}{\partial\theta^2}$$

In compartment 2 ($L_1 + L \le \theta \le L_1 + L + L_2 = l$),

$$\frac{\partial[X]}{\partial t} = k_4[C_2] + D_X\frac{\partial^2[X]}{\partial\theta^2}$$
$$\frac{\partial[X^*]}{\partial t} = -k_3[X^*][E_2] + k_{-3}[C_2] - k_5[X^*][Y] + k_{-5}[C_3] + k_6[C_3] + D_X\frac{\partial^2[X^*]}{\partial\theta^2}$$
$$\frac{\partial[Y]}{\partial t} = -k_5[X^*][Y] + k_{-5}[C_3] + k_8[C_4]$$
$$\frac{\partial[Y^*]}{\partial t} = -k_7[Y^*][E_3] + k_{-7}[C_3] + k_6[C_4]$$
$$\frac{\partial[C_2]}{\partial t} = k_3[X^*][E_2] - k_{-3}[C_2] - k_4[C_2]$$
$$\frac{\partial[C_3]}{\partial t} = k_5[X^*][Y] - k_{-5}[C_3] - k_6[C_3]$$
$$\frac{\partial[C_4]}{\partial t} = k_7[Y^*][E_3] - k_{-7}[C_4] - k_8[C_4]$$

With the conservation equations,

$$X^{Total} = (\langle[X]\rangle + \langle[X^*]\rangle)l + \langle[C_1]\rangle_1 L_1 + \langle[C_2]\rangle_1 L_1 + \langle[C_3]\rangle_1 L_2,$$
$$Y^{Total} = (\langle[Y]\rangle_2 + \langle[Y^*]\rangle_2)L_2 + \langle[C_3]\rangle_2 L_2 + \langle[C_4]\rangle_2 L_2,$$
$$E_1^{Total} = (\langle[E_1]\rangle_1 + \langle[C_1]\rangle_1)L_1$$
$$E_2^{Total} = (\langle[E_2]\rangle_2 + \langle[C_2]\rangle_2)L_2$$
$$E_3^{Total} = (\langle[E_3]\rangle_2 + \langle[C_4]\rangle_2)L_2$$

The corresponding compartmental model is as follows.

Compartmental ODE model: In compartment 1,

$$L_1 \frac{d[X_1]}{dt} = -k_1[X_1][E_1]L_1 + k_{-1}[C_1]L_1 - tr^X_{1\to 2}[X_1]L_1 + tr^X_{2\to 1}[X_2]L_2$$

$$L_1 \frac{d[X_1^*]}{dt} = k_2[C_1]L_1 - tr^{X^*}_{1\to 2}[X_1^*]L_1 + tr^{X^*}_{2\to 1}[X_2^*]L_2$$

$$\frac{d[C_1]}{dt} = k_1[X_1][E_1] - k_{-1}[C_1] - k_2[C_1]$$

In compartment 2 ($L_1 + L \leq \theta \leq L_1 + L + L2$),

$$L_2 \frac{d[X_2]}{dt} = k_4[C_2]L_2 - tr^X_{2\to 1}[X_2]L_2 + tr^X_{1\to 2}[X_1]L_1$$

$$L_2 \frac{d[X_2^*]}{dt} = -k_3[X_1^*][E_2]L_2 + k_{-3}[C_2]L_2 - k_5[X_2^*][Y]L_2 + k_{-5}[C_3]L_2 + k_6[C_3] - tr^{X^*}_{2\to 1}[X_2^*]L_2 + $$ [illegible]

$$\frac{d[Y]}{dt} = -k_5[X_2^*][Y] + k_{-5}[C_3] + k_8[C_4]$$

$$\frac{d[Y^*]}{dt} = -k_7[Y^*][E_3] + k_{-7}[C_3] + k_6[C_4]$$

$$\frac{d[C_2]}{dt} = k_3[X_1^*][E_2] - k_{-3}[C_2] - k_4[C_2]$$

$$\frac{d[C_3]}{dt} = k_5[X_2^*][Y] - k_{-5}[C_3] - k_6[C_3]$$

$$\frac{d[C_4]}{dt} = k_7[Y^*][E_3] - k_{-7}[C_4] - k_8[C_4]$$

With corresponding conservation equations for the substrates and the enzymes.

1.3 Two-site modification

This is a closed system involving interconversion between the species X and its modified forms, X^* and X^{**}, with the three forms belonging to a conserved pool. As in the previous models, both enzymes and complexes are non-diffusing. Enzyme E_1, uniformly distributed in compartment 1, catalyzes both the forward steps $X \to X^*$ and $X^* \to X^{**}$, while E_2, uniformly distributed in compartment 2, catalyzes both the reverse steps.

PDE model: In compartment 1 ($0 \le \theta \le L_1$),

$$\begin{aligned}
\frac{\partial[X]}{\partial t} &= -k_1[X][E_1] + k_{-1}[C_1] + D_X \frac{\partial^2[X]}{\partial\theta^2} \\
\frac{\partial[X^*]}{\partial t} &= -k_3[X^*][E_1] + k_{-3}[C_2] + k_2[C_1] + D_{X^*} \frac{\partial^2[X^*]}{\partial\theta^2} \\
\frac{\partial[X^{**}]}{\partial t} &= k_4[C_2] + D_{X^{**}} \frac{\partial^2[X^{**}]}{\partial\theta^2} \\
\frac{\partial[C_1]}{\partial t} &= k_1[X][E_1] - k_{-1}[C_1] - k_2[C_1] \\
\frac{\partial[C_2]}{\partial t} &= k_3[X^*][E_1] - k_{-3}[C_2] - k_4[C_2]
\end{aligned}$$

Between the compartments ($L_1 \le \theta \le L_1 + L$),

$$\begin{aligned}
\frac{\partial[X]}{\partial t} &= D_X \frac{\partial^2[X]}{\partial\theta^2} \\
\frac{\partial[X^*]}{\partial t} &= D_{X^*} \frac{\partial^2[X^*]}{\partial\theta^2} \\
\frac{\partial[X^{**}]}{\partial t} &= D_{X^{**}} \frac{\partial^2[X^{**}]}{\partial\theta^2}
\end{aligned}$$

In compartment 2 ($L_1 + L \le \theta \le L_1 + L + L_2 = l$),

$$\begin{aligned}
\frac{\partial[X]}{\partial t} &= k_8[C_4] + D_X \frac{\partial^2[X]}{\partial\theta^2} \\
\frac{\partial[X^*]}{\partial t} &= -k_7[X^*][E_2] + k_{-7}[C_4] + k_6[C_3] + D_{X^*} \frac{\partial^2[X^*]}{\partial\theta^2} \\
\frac{\partial[X^{**}]}{\partial t} &= -k_5[X^{**}][E_2] + k_{-5}[C_3] + D_{X^{**}} \frac{\partial^2[X^{**}]}{\partial\theta^2} \\
\frac{\partial[C_3]}{\partial t} &= k_5[X^{**}][E_2] - k_{-5}[C_3] - k_6[C_3] \\
\frac{\partial[C_4]}{\partial t} &= k_7[X^*][E_2] - k_{-7}[C_4] - k_8[C_4]
\end{aligned}$$

With the conservation equations,

$$\begin{aligned}
X^{Total} &= (\langle[X]\rangle + \langle[X^*]\rangle + \langle[X^{**}]\rangle)l + \langle[C_1]\rangle_1 L_1 + \langle[C_2]\rangle_1 L_1 + \langle[C_3]\rangle_1 L_2 + \langle[C_4]\rangle_1 L_2, \\
E_1^{Total} &= (\langle[E_1]\rangle_1 + \langle[C_1]\rangle_1 + \langle[C_2]\rangle_1)L_1 \\
E_2^{Total} &= (\langle[E_2]\rangle_1 + \langle[C_3]\rangle_2 + \langle[C_4]\rangle_2)L_2
\end{aligned}$$

The corresponding compartmental model is as follows.

Compartmental ODE model: In compartment 1,

$$L_1 \frac{d[X]}{dt} = -k_1[X_1][E_1]L_1 + k_{-1}[C_1]L_1 - tr^{X}_{1\to 2}[X_1]L_1 + tr^{X}_{2\to 1}[X_2]L_2$$

$$\begin{aligned}
L_1 \frac{d[X_1^*]}{dt} &= -k_3[X_1^*][E_1]L_1 + k_{-3}[C_2]L_1 + k_2[C_1]L_1 - tr^{X^*}_{1\to 2}[X_1^*]L_1 + tr^{X^*}_{2\to 1}[X_2^*]L_2 \\
L_1 \frac{d[X_1^{**}]}{dt} &= k_4[C_2]L_1 - tr^{X^{**}}_{1\to 2}[X_1^{**}]L_1 + tr^{X^{**}}_{2\to 1}[X_2^{**}]L_2 \\
\frac{d[C_1]}{dt} &= k_1[X_1][E_1] - k_{-1}[C_1] - k_2[C_1] \\
\frac{d[C_2]}{dt} &= k_3[X_1^*][E_1] - k_{-3}[C_2] - k_4[C_2]
\end{aligned}$$

In compartment 2,

$$\begin{aligned}
L_2 \frac{d[X_2]}{dt} &= k_8[C_4]L_2 - tr^{X}_{2\to 1}[X_2]L_2 + tr^{X}_{1\to 2}[X_1]L_1 \\
L_2 \frac{d[X_2^*]}{dt} &= -k_7[X_2^*][E_2]L_2 + k_{-7}[C_2]L_2 + k_6[C_3]L_2 - tr^{X^*}_{2\to 1}[X_2^*]L_2 + tr^{X^*}_{1\to 2}[X_1^*]L_1 \\
L_2 \frac{d[X_2^{**}]}{dt} &= -k_5[X_2^{**}][E_2]L_2 + k_{-5}[C_3]L_2 - tr^{X^{**}}_{2\to 1}[X_2^{**}]L_2 + tr^{X^{**}}_{1\to 2}[X_1^{**}]L_1 \\
\frac{d[C_3]}{dt} &= k_5[X_2^{**}][E_2] - k_{-5}[C_3] - k_6[C_3] \\
\frac{d[C_4]}{dt} &= k_7[X_2^*][E_2] - k_{-7}[C_4] - k_8[C_4]
\end{aligned}$$

With corresponding conservation equations for the substrates and the enzymes.

1.4 Simple open system

This is an open system involving a single species X that is produced in compartment 1 and degraded in compartment 2.

PDE model: In compartment 1 ($0 \le \theta \le L_1$),

$$\frac{\partial[X]}{\partial t} = k_0 + D_X \frac{\partial^2[X]}{\partial\theta^2}$$

Between the compartments ($L_1 \le \theta \le L_1 + L$),

$$\frac{\partial[X]}{\partial t} = D_X \frac{\partial^2[X]}{\partial\theta^2}$$

In compartment 2 ($L_1 + L \leq \theta \leq L_1 + L + L_2 = l$),

$$\frac{\partial[X]}{\partial t} = -k_1[X] + D_X \frac{\partial^2[X]}{\partial\theta^2}$$

The corresponding compartmental model is as follows.

Compartmental ODE model: In compartment 1,

$$L_1 \frac{d[X]}{dt} = k_0 L_1 - tr^X_{1\to2}[X_1]L_1 + tr^X_{2\to1}[X_2]L_2$$

In compartment 2,

$$L_2 \frac{d[X_2]}{dt} = -k_1[X_2]L_2 - tr^X_{2\to1}[X_2]L_2 + tr^X_{1\to2}[X_1]L_1$$

1.5 Metabolic pathway

This system consists of part of a metabolic pathway, involving two successive enzymatic conversions of metabolites. The two enzymes, as well as the initial metabolite R, are non-diffusible, while the product of the first conversion X, is diffusible across the whole domain. R is present in compartment 1, with the first enzyme P also confined to this compartment. The concentration of R is assumed to be maintained at a fixed level in compartment 1. The second enzyme E, that converts X to another metabolite Y, is distributed between the two compartments (uniformly in each compartment), with a possible difference in its kinetics in compartments 1 and 2 . There are competing reactions, consuming X, in the second compartment. The enzymatic reactions are assumed to operate at the mass-action regime.

PDE model: In compartment 1 ($0 \leq \theta \leq L_1$),

$$\frac{\partial[X]}{\partial t} = k_1[P][R] - k_{21}[E][X] + D_X \frac{\partial^2[X]}{\partial\theta^2}$$

Between the compartments ($L_1 \leq \theta \leq L_1 + L$),

$$\frac{\partial[X]}{\partial t} = D_X \frac{\partial^2[X]}{\partial\theta^2}$$

In compartment 2 ($L_1 + L \leq \theta \leq L_1 + L + L_2 = l$),

$$\frac{\partial[X]}{\partial t} = -k_{22}[E][X] - k_3[X] + D_X \frac{\partial^2[X]}{\partial\theta^2}$$

The total amount of the enzyme F is fixed,

$$E^{Total} = \langle[E]\rangle_1 L_1 + \langle[E]\rangle_2 L_2$$

The corresponding compartmental model is as follows.

Compartmental ODE model: In compartment 1,

$$L_1 \frac{d[X_1]}{\partial t} = k_1[P][R]L_1 - k_{21}[E_1][X]L_1 - tr^X_{1\to2}[X_1]L_1 + tr^X_{2\to1}[X_2]L_2$$

In compartment 2,

$$L_2 \frac{d[X]}{dt} = -k_{22}[E_2][X]L_2 - k_3[X]L_2 - tr^X_{1\to2}[X_1]L_1 + tr^X_{2\to1}[X_2]L_2,$$

with the total amount of the enzyme E,

$$E^{Total} = [E_1]L_1 + [E_2]L_2$$

We also examine a variant of the above pathway, where the product metabolite X negatively regulates the enzyme E. This type of negative feedback by a metabolite on the upstream enzymes in a pathway may be realised through allosteric regulation. In this case, the production term for species X in compartment 1 is changed to $\frac{k_1[P][R]}{K+[X]}$ in the PDE and $\frac{k_1[P][R]}{K+[X]}L_1$ in the compartmental model.

2 Analytical Investigations

In this section, we present a series of investigations to consolidate and further discuss points made in the main text. This includes solution of the relevant PDEs, correspondence with the relevant compartmental models (and obtaining relevant parameters therein, as well as other related discussions). In all cases, analytical solutions to the PDE are cross-validated against numerical solutions. Furthermore, parametrizing or modifying compartmental models to account for sequestration or changing transport parameters is done through a correspondence between compartmental ODE models and the PDE. This is subject to an additional level of cross-validation. Indeed, as discussed in the main text, these changes have demonstrated a marked improvement in matching the compartmental ODE model with the PDE in many different parameter regimes and scenarios. Numerical evidence in the text substantiates other analytical discussions.

2.1 Transport parameters in the thin compartment regime

For the kinds of compartmentalized reaction pathways examined in this study, compartmental ODE models naturally correspond to the limit of well mixed compartments connected by pure diffusive transport. This limit is usually assumed when using compartmental ODE models. This limit may be

realised in different ways - for the type of scenario examined here, this limit corresponds to having compartment sizes much smaller than the intervening space, i.e. the thin compartment regime.

Consider any 1-D, two compartment system (the following discussion is equally applicable to systems with more compartments) of the kind examined here (for example, see Figure 1). The net transport rate of a species X_j out of compartment 1 may be expressed as the product of the diffusivity D_j and the concentration gradient of X_j at the edge of the compartment. Since the intervening space involves only diffusion, this transport rate, at the limit of well mixed compartments, is given by

$$D_j \frac{X_{j1} - X_{j2}}{L}, \tag{1}$$

where X_{j1} and X_{j2} are the concentrations of X_j in compartments 1 and 2, and L is the size of the space between compartments. Now, the net transport rate of the same species X out of compartment 1, in the corresponding compartmental ODE model is represented as

$$L_1 tr^X_{1\to 2} X_{j1} - L_2 tr^X_{2\to 1} X_{j2}. \tag{2}$$

From (1) and (2), we see that the transport parameters for X corresponding to the limit of well mixed compartments is given by

$$tr^X_{1\to 2} = \frac{D_X}{LL_1}$$
$$tr^X_{2\to 1} = \frac{D_X}{LL_2}.$$

In the following discussion, we use analytical solutions to the PDE (for the simple closed and open systems) to obtain expressions for the transport parameters, and show that the above values for the transport parameters are attained in the limit of thin compartments.

2.2 Accounting for sequestration: Modified conservation condition

Consider the simple closed system, the compartmentalized single modification cycle discussed in the text (see Figure 1(a)). In the thin compartment regime, the total amount of substrate in the compartments depends on kinetics, while the transport parameters may be approximated by D/LL_1. In order for such a compartmental ODE model to completely capture the effect

of changing kinetics, it needs a way of accounting for the variation in total amount of substrate (in the compartments).

Now, although the total amount in the compartments, i.e. $\tilde{X}^{Total}$, may depend on kinetics, the total amount in the whole domain is still conserved and remains invariant. That is,

$$X^{Total} = \tilde{X}^{Total} + X^{Inter}$$

is fixed independent of the kinetics, where X^{Inter} represents the total amount of X and X^* in the intermediate space. Note that the enzyme-substrate complexes, being non-diffusible, are not present in the intermediate space. Since the concentration profiles of X and X^* are linear in the intermediate space (due to pure diffusion in the intermediate space), X^{Inter} can be computed exactly using the concentrations of X and X^* at the edges of the compartments (i.e. at $\theta = L_1$ and $\theta = L_1 + L$).

$$X^{Inter} = \frac{X(L_1) + X(L_2)}{2} L + \frac{X^*(L_1) + X^*(L_2)}{2} L$$

In the thin compartment regime, in the absence of significant gradients within the compartments, these edge concentrations may be reasonably approximated by the compartmental averages. This means that, for the compartmental ODE, we can write a conservation condition for the whole domain:

$$\begin{aligned} X^{Total} = & [X_1]L_1 + [X_2]L_2 + [X_1^*]L_1 + [X_2^*]L_2 + [C_1]L_1 + [C_2]L_2 \\ & + \frac{[X_1] + [X_2]}{2} L + \frac{[X_1^*] + [X_2^*]}{2} L \end{aligned}$$

For the single modification cycle, there is only one enzyme-substrate complex in each compartment - C_1 in compartment 1 and C_2 in compartment 2.

The above conservation condition involves only the compartmental variables, but still accounts for the sequestration of species in the intermediate space. Note that we have explicitly used steady state conditions in obtaining this conservation condition.

2.3 Accounting for sequestration: Using a third compartment for the intermediate space

The modified conservation condition described above approximates the total amount in the intermediate space well, for a system in the thin compartment regime. However, this approximation only describes the steady state. Away from steady state conditions, the total amount in the intermediate space may

not be equal to the average of the compartmental concentrations multiplied by the size of the intermediate space. One way to overcome this limitation, is to use a third compartment to describe the intermediate space.

Consider a general pathway involving two compartments in 1-D. In the thin compartment regime, the basic (two) compartmental model is given by:

$$\begin{aligned} L_1\frac{dX_{j1}}{dt} &= L_1 f_{j1}(X_{11}, X_{21}, ..., X_{n1}) - L_1 tr^j_{1\to 2}X_{j1} + L_2 tr^j_{2\to 1}X_{j2} \\ L_2\frac{dX_{j2}}{dt} &= L_2 f_{j2}(X_{12}, X_{22}, ..., X_{n2}) - L_2 tr^j_{2\to 1}X_{j2} + L_1 tr^j_{1\to 2}X_{j1} \end{aligned} \tag{3}$$

$$\text{where } j = 1, 2, ..., n$$

where the variables X_{ji} represent the (average) concentration of species j in compartment i. The reaction kinetic terms are represented by the functions $f_{ji}(X_{1i}, X_{2i}, ..., X_{ni})$. Each species j has a pair of transport parameters associated with it - $tr^j_{1\to 2}$ and $tr^j_{1\to 2}$. As seen above, in the thin compartment regime, these are well approximated by $\frac{D_j}{LL_1}$ and $\frac{D_j}{LL_2}$ respectively, where D_j is the diffusivity of species j. Thus, the transport rate of X_j out of compartment 1 (into compartment 2) has the form (1).

Now, instead of just using a modified conservation condition for each diffusing species that is conserved, we introduce a third compartment representing the intermediate space. The compartmental model is now given by

$$\begin{aligned} L_1\frac{dX_{j1}}{dt} &= L_1 f_{j1}(X_{11}, X_{21}, ..., X_{n1}) - L_1 tr^j_{1\to m}X_{j1} + L tr^j_{m\to 1}X_{jm} \\ L\frac{dX_{jm}}{dt} &= L_1 tr^j_{1\to m}X_{j1} - L tr^j_{m\to 1}X_{jm} + L_2 tr^j_{2\to m}X_{j2} - L tr^j_{m\to 2}X_{jm} \\ L_2\frac{dX_{j2}}{dt} &= L_2 f_{j2}(X_{12}, X_{22}, ..., X_{n2}) - L_2 tr^j_{2\to m}X_{j2} + L tr^j_{m\to 2}X_{jm} \end{aligned} \tag{4}$$

$$\text{where } j = 1, 2, ..., n$$

Here, X_{jm} represents the (average) concentration of species j in the intermediate space denoted by m. Suppose that transport parameters are chosen

in the following way:

$$tr^j_{1\to m} = \frac{2D_j}{LL_1},$$
$$tr^j_{2\to m} = \frac{2D_j}{LL_2},$$
$$tr^j_{m\to 1} = tr^j_{m\to 2} = \frac{2D_j}{L^2}.$$

At steady state, this gives $X_{jm} = \frac{X_{j1}+X_{j2}}{2}$, and the transport rate of X_j out of compartment 1 (into compartment 2) continues to have the form (1). Now, the conservation condition for a conserved, diffusing species may be correctly approximated by incorporating the corresponding amount in the third compartment, when computing the total amount of substrate. Note, that the above model fails completely if any reactions involving net production or removal of X_j are present in the intermediate space, even if the corresponding kinetic terms are incorporated into (4). This is because the resulting system would no longer correspond to the limit of thin compartments.

Now we discuss analytical solutions to the PDE models for the single modification cycle and the simple open system discussed in the main text.

2.4 Solutions to PDE models

Case 1: Single modification cycle(mass-action)

1-D PDE model:

We assume that both phosphorylation and dephosphorylation occure through mass action kinetics. Note that this can be formally obtained from the model in the previous section by making the catalytic constants high. The kinase and phosphatase are uniformly distributed in the regions $0 \le \theta < L_1$ and $L_1 + L \le \theta < l < L_1 + L + L_2$ respectively (see Figure 1(a)). We assume that both the phosphorylation and dephosphorylation involve mass action kinetics. We first consider the case where X and X^* are equally diffusible.

In compartment 1 ($0 \le \theta \le L_1$),

$$\frac{\partial [X]}{\partial t} = -k_1[X] + D\frac{\partial^2 [X]}{\partial \theta^2}$$
$$\frac{\partial [X^*]}{\partial t} = k_1[X] + D\frac{\partial^2 [X^*]}{\partial \theta^2}$$

Between the compartments ($L_1 \leq \theta \leq L_1 + L$),

$$\frac{\partial[X]}{\partial t} = D\frac{\partial^2[X]}{\partial\theta^2}$$
$$\frac{\partial[X^*]}{\partial t} = D\frac{\partial^2[X^*]}{\partial\theta^2}$$

In compartment 2 ($L_1 + L \leq \theta \leq L_1 + L + L_2 = l$),

$$\frac{\partial[X]}{\partial t} = k_2[X^*] + D\frac{\partial^2[X]}{\partial\theta^2}$$
$$\frac{\partial[X^*]}{\partial t} = -k_2[X^*] + D\frac{\partial^2[X^*]}{\partial\theta^2}$$

With the conservation condition,

$$X^{Total} = (\langle[X]\rangle + \langle[X^*]\rangle)l,$$

where $\langle\cdot\rangle$ represents the spatial average over the whole domain. In the above, k_1 represents the product of the phosphorylation rate constant and the total kinase concentration, while k_2 represents the product of the dephosphorylation rate constant and the total phosphatase concentration

Since X and X^* are equally diffusible, the PDE model implies that the quantity $[X]+[X^*]$ must be constant throughout the domain at steady state. That is, $[X] + [X^*] = X_T$, where $X_T = X^{Total}/l$. This also means that the total amount of substrate sequestered in the middle is independent of the kinetic parameters in the compartments. Also note that the equations for X and X^* are linear. Combined with the above condition, this means that $[X]$ and $[X^*]$ are linearly proportional to X_T (and hence to X^{Total}).

1-D PDE Solution

Implementing no-flux boundary conditions we find:

$$\begin{aligned}
For\ 0 \leq \theta < L_1 :\ [X] &= c_1 X_T(e^{\omega_1\theta} + e^{-\omega_1\theta}) \\
[X^*] &= X_T(1 - c_1(e^{\omega_1\theta} + e^{-\omega_1\theta})) \\
For\ L_1 \leq \theta < L_1 + L :\ [X] &= X_T(c_2\theta + c_3) \\
[X^*] &= X_T(1 - c_2\theta - c_3) \\
For\ L_1 + L \leq \theta < l :\ [X] &= c_4 X_T(e^{\omega_2\theta} + e^{\omega_2(2l-\theta)}) \\
[X^*] &= X_T(1 - c_4(e^{\omega_2\theta} + e^{\omega_2(2l-\theta)})) \\
where\ l = L_1 + L + L_2;\ \omega_1^2 &= \frac{k_1}{D};\ \omega_2^2 = \frac{k_2}{D}
\end{aligned}$$

$$
\begin{aligned}
c_1 &= \frac{1}{(e^{\omega_1 L_1} + e^{-\omega_1 L_1}) + \omega_1 L(e^{\omega_1 \theta} - e^{-\omega_1 \theta}) - \frac{\omega_1 (e^{\omega_1 \theta} - e^{-\omega_1 \theta})(e^{\omega_2 (L_1 + L)} + e^{\omega_2 (2l - L_1 - L)})}{\omega_2 (e^{\omega_2 (L_1 + L)} - e^{\omega_2 (2l - L_1 - L)})}} \\
c_2 &= c_1 \omega_1 (e^{\omega_1 \theta} - e^{-\omega_1 \theta}) \\
c_3 &= c_1 (e^{\omega_1 L_1} + e^{-\omega_1 L_1}) - c_2 L \\
c_4 &= \frac{-c_2}{\omega_2 (e^{\omega_2 (L_1 + L)} - e^{\omega_2 (2l - L_1 - L)})}
\end{aligned}
$$

Compartmental averages

$$
\begin{aligned}
\langle X \rangle_1 &= \frac{c_1 X_T}{\omega_1 L_1} (e^{\omega_1 L_1} - e^{-\omega_1 L_1}) \\
\langle X^* \rangle_2 &= \frac{c_4 X_T}{\omega_2 L_2} (e^{\omega_2 (L_1 + L)} - e^{\omega_2 (2l - L_1 - L)})
\end{aligned} \tag{5}
$$

Since the diffusivities of X and X^* are equal, the compartmental averages must also satisfy the equations

$$
\begin{aligned}
\langle X \rangle_1 + \langle X^* \rangle_1 &= X_T \\
\langle X \rangle_2 + \langle X^* \rangle_2 &= X_T
\end{aligned} \tag{6}
$$

We now consider the corresponding compartmental ODE model for the case where the volumes of the two compartments are equal, i.e. $L_1 = L_2$. The case where $L_1 \neq L_2$ is essentially no different. Since the volumes are equal, we have a single transport parameter associated with each species - tr^X for X and tr^{X^*} for X^*. The model is as follows.

In compartment 1,

$$
\begin{aligned}
\frac{d[X_1]}{dt} &= -k_1[X_1] - tr^X[X_1] + tr^X[X_2] \\
\frac{d[X_1^*]}{dt} &= k_1[X_1] - tr^{X^*}[X_1^*] + tr^{X^*}[X_2^*]
\end{aligned}
$$

In compartment 2,

$$
\begin{aligned}
\frac{d[X_2]}{dt} &= k_2[X_2^*] - tr^X[X_2] + tr^X[X_1] \\
\frac{d[X_2^*]}{dt} &= -k_3[X_2^*] - tr^{X^*}[X_2^*] + tr^{X^*}[X_1^*]
\end{aligned} \tag{7}
$$

Transport parameter

The values of the transport parameters that make the steady state of the compartmental model exactly match the PDE averages may then be computed as follows:

$$
\begin{aligned}
tr^{X} &= \frac{k_1 \langle X \rangle_1}{\langle X \rangle_2 - \langle X \rangle_1} \\
tr^{X^*} &= \frac{-k_1 \langle X \rangle_1}{\langle X^* \rangle_2 - \langle X^* \rangle_1}
\end{aligned}
$$

As we show below, the two transport parameters must be in the same ratio as the diffusivities of X and X^*. Thus, for the case of equal diffusivities, we have a single transport parameter tr. Using equations (5) and (6), we have

$$
tr = \frac{k_1 \frac{c_1}{\omega_1 L_1}(e^{\omega_1 L_1} - e^{-\omega_1 L_1})}{1 - (1 + \frac{k_1}{k_2})\frac{c_1}{\omega_1 L_1}(e^{\omega_1 L_1} - e^{-\omega_1 L_1})} \tag{8}
$$

Taking the limit $L \to \infty$, keeping $\frac{D}{L}$ constant, we find that

$$
\begin{aligned}
\lim_{L\to\infty} \langle X \rangle_1 = \lim_{L\to\infty} 2c_1 X_T &= \frac{X_T}{\left(1 + \frac{k_1}{k_2} + \frac{k_1 L L_1}{D}\right)} \\
\implies \lim_{L\to\infty} tr &= \frac{D}{L_1 L}
\end{aligned}
$$

This shows how the expression D/LL_1 can be reached naturally from the expression for the transport parameter.

We now examine the case where X and X^* have different diffusivities, D_X and D_{X^*}. To find the steady state solution to this system, we use a change of variables: $[\widetilde{X}] = D_X[X]$ and $[\widetilde{X}^*] = D_{X^*}[X^*]$. At steady state, this gives us a system of the same form as the equal diffusivity case (with $D = 1$), in the new variables $[\widetilde{X}]$ and $[\widetilde{X}^*]$. Note that, in the new system, the rate constants multiplying $[\widetilde{X}]$ and $[\widetilde{X}^*]$ are $\frac{k_1}{D_X}$ and $\frac{k_2}{D_{X^*}}$ respectively. Now, we can use the solution previously obtained in the equal diffusivity case, with these rate constants, and setting $D = 1$, to compute the profiles of the new variables $[\widetilde{X}]$ and $[\widetilde{X}^*]$. The actual profiles are then given by $[X] = \frac{[\widetilde{X}]}{D_X}$ and $[X^*] = \frac{[\widetilde{X}^*]}{D_{X^*}}$.

The compartmental model for the system is given by (7). The values of the transport parameters that produce an exact match with the PDE

averages at steady state are computed as follows:

$$tr^X = \frac{k_1 \langle X \rangle_1}{\langle X \rangle_2 - \langle X \rangle_1} = \frac{k_1 \frac{\langle \widetilde{X} \rangle_1}{D_X}}{\frac{1}{D_X}\left(\langle \widetilde{X} \rangle_2 - \langle \widetilde{X} \rangle_1\right)} = \frac{k_1 \langle \widetilde{X} \rangle_1}{\langle \widetilde{X} \rangle_2 - \langle \widetilde{X} \rangle_1} \tag{9}$$

$$tr^{X^*} = \frac{-k_1 \langle X \rangle_1}{\langle X^* \rangle_2 - \langle X^* \rangle_1} = \frac{-k_1 \frac{\langle \widetilde{X} \rangle_1}{D_X}}{\frac{1}{D_{X^*}}\left(\langle \widetilde{X}^* \rangle_2 - \langle \widetilde{X}^* \rangle_1\right)} = \frac{k_1 \langle \widetilde{X} \rangle_1}{\langle \widetilde{X} \rangle_2 - \langle \widetilde{X} \rangle_1} \left(\frac{D_{X^*}}{D_X}\right), \tag{10}$$

where we have also used the fact that $\langle \widetilde{X} \rangle$ and $\langle \widetilde{X}^* \rangle$ satisfy equations of the form (6). From (9) and (10), we see that the transport parameters are in the same ratio as the diffusivities.

Case 2: Simple open system

This is an open system involving a single species X that is produced in compartment 1 (rate constant k_0) and degraded/consumed in compartment 2 (rate constant k_1).

1-D PDE Model

In compartment 1 ($0 \leq \theta \leq L_1$),

$$\frac{\partial [X]}{\partial t} = k_0 + D_X \frac{\partial^2 [X]}{\partial \theta^2}$$

Between the compartments ($L_1 \leq \theta \leq L_1 + L$),

$$\frac{\partial [X]}{\partial t} = D_X \frac{\partial^2 [X]}{\partial \theta^2}$$

In compartment 2 ($L_1 + L \leq \theta \leq L_1 + L + L_2 = l$),

$$\frac{\partial [X]}{\partial t} = -k_1 [X] + D_X \frac{\partial^2 [X]}{\partial \theta^2}$$

1-D PDE Solution

The solution is as follows:

$$For\ 0 \leq \theta < L_1:\ X = k_0\left(\frac{-\theta^2}{2D} + c_2\right)$$

$$For\ L_1 \leq \theta < L_1 + L:\ X = k_0(c_3\theta + c_4)$$

$$For\ L_1 + L \leq \theta < l:\ X = k_0 c_5(e^{\omega_1\theta} + e^{\omega_1(2l-\theta)})$$

$$where\ l = L_1 + L + L_2;\ \omega_1^2 = \frac{k_1}{D};$$

$$\begin{aligned}
c_2 &= \left(\frac{L_1^2}{2D} + \frac{k_0 L_1 L}{D} - \frac{k_0 L_1 (e^{\omega_1(L_1+L)} + e^{\omega_1(2l-L_1-L)})}{\omega_1 D(e^{\omega_1(L_1+L)} - e^{\omega_1(2l-L_1-L)})}\right)\\
c_3 &= \frac{-L_1}{D}\\
c_4 &= \frac{L_1^2}{D} + \frac{L_1 L}{D} - \frac{L_1(e^{\omega_1(L_1+L)} + e^{\omega_1(2l-L_1-L)})}{\omega_1 D(e^{\omega_1(L_1+L)} - e^{\omega_1(2l-L_1-L)})}\\
c_5 &= \frac{-L_1}{\omega_1 D(e^{\omega_1(L_1+L)} - e^{\omega_1(2l-L_1-L)})}
\end{aligned}$$

Compartmental averages

$$\begin{aligned}
\langle X\rangle_1 &= k_0\left(\frac{-L_1^2}{6D} + c_2\right)\\
\langle X\rangle_2 &= k_0\left(\frac{-c_5(e^{\omega_1(L_1+L)} - e^{\omega_1(2l-L_1-L)})}{\omega_1 L_2}\right) = \frac{k_0 L_1}{k_1 L_2}
\end{aligned} \qquad (11)$$

We now consider the corresponding compartmental model for the case where the volumes of the two compartments are equal, i.e. $L_1 = L_2$. The case where $L_1 \neq L_2$ is essentially no different. Since the volumes are equal, we have a single transport parameter associated the species X. The model is given by:

Compartmental ODE model: In compartment 1,

$$\frac{d[X]}{dt} = k_0 - tr^X[X_1] + tr^X[X_2]$$

In compartment 2,

$$\frac{d[X_2]}{dt} = -k_1[X_2] - tr^X[X_2] + tr^X[X_1]$$

Transport parameter

The values of the transport parameters that make the steady state of the compartmental model exactly match the PDE averages may then be computed as follows:

$$tr^X = \frac{k_0}{\langle X\rangle_1 - \langle X\rangle_2}, \tag{12}$$

where $\langle X\rangle_1$ and $\langle X\rangle_2$ are given by (11). Substituting for $\langle X\rangle_1$ and $\langle X\rangle_2$ and taking the limit $L \to \infty$, keeping $\frac{D}{L}$ constant, we find that

$$\lim_{L\to\infty} \langle X\rangle_1 = \lim_{L\to\infty} k_0 c_2 = k_0 L_1 \left(\frac{1}{k_1 L_2} + \frac{L}{D} \right)$$
$$\implies \lim_{L\to\infty} tr^X = \frac{D}{L_1 L}$$

Case 3: Simple open system (with degradation in both compartments)

This is an open system involving a single species X that is produced in compartment 1 (rate constant k_0) and degraded/consumed in both compartment 1 (rate constant k_1) and compartment 2 (rate constant k_2).

1-D PDE Model

In compartment 1 ($0 \leq \theta \leq L_1$),

$$\frac{\partial[X]}{\partial t} = k_0 - k_1[X] + D_X \frac{\partial^2[X]}{\partial\theta^2}$$

Between the compartments ($L_1 \leq \theta \leq L_1 + L$),

$$\frac{\partial[X]}{\partial t} = D_X \frac{\partial^2[X]}{\partial\theta^2}$$

In compartment 2 ($L_1 + L \leq \theta \leq L_1 + L + L_2 = l$),

$$\frac{\partial[X]}{\partial t} = -k_2[X] + D_X \frac{\partial^2[X]}{\partial\theta^2}$$

1-D PDE Solution

The solution is as follows:

$$For\ 0 \leq \theta < L_1 :\ X = k_0 \left(c_1(e^{\omega_1\theta} + e^{-\omega_1\theta}) + \frac{1}{k_1} \right)$$

$$For\ L_1 \leq \theta < L_1 + L :\ X = k_0(c_3\theta + c_4)$$

$$For\ L_1 + L \leq \theta < l :\ X = k_0 c_5(e^{\omega_2\theta} + e^{\omega_2(2l-\theta)})$$

$$where\ l = L_1 + L + L_2;\ \omega_1^2 = \frac{k_1}{D};\ \omega_2^2 = \frac{k_2}{D};$$

$$\begin{aligned}
c_3 &= \frac{1}{k_1\left(\frac{(e^{\omega_2(L_1+L)}+e^{\omega_2(2l-L_1-L)})}{\omega_2(e^{\omega_2(L_1+L)}-e^{\omega_2(2l-L_1-L)})} - \frac{(e^{\omega_1 L_1}+e^{-\omega_1 L_1})}{\omega_1(e^{\omega_1 L_1}-e^{-\omega_1 L_1})} - L\right)} \\
c_1 &= \frac{c_3}{\omega_1(e^{\omega_1 L_1} - e^{-\omega_1 L_1})} \\
c_4 &= c_1(e^{\omega_1 L_1} + e^{-\omega_1 L_1}) + \frac{1}{k_1} - c_3 L_1 \\
c_5 &= \frac{c_3}{\omega_2(e^{\omega_2(L_1+L)} - e^{\omega_2(2l-L_1-L)})}
\end{aligned}$$

Compartmental averages

$$\begin{aligned}
\langle X \rangle_1 &= k_0 \left(\frac{c_1}{\omega_1 L_1}(e^{\omega_1 L_1} - e^{-\omega_1 L_1}) + \frac{1}{k_1} \right) \\
\langle X \rangle_2 &= k_0 \left(\frac{c_5}{\omega_2 L_2}(e^{\omega_2(L_1+L)} - e^{\omega_2(2l-L_1-L)}) \right)
\end{aligned}$$

We now consider the corresponding compartmental model for the case where the volumes of the two compartments are equal, i.e. $L_1 = L_2$. The case where $L_1 \neq L_2$ is essentially no different. Since the volumes are equal, we have a single transport parameter associated the species X. The model is given by:

Compartmental ODE model: In compartment 1,

$$\frac{d[X]}{dt} = k_0 - k_1[X_1] - tr^X[X_1] + tr^X[X_2]$$

In compartment 2,

$$\frac{d[X_2]}{dt} = -k_2[X_2] - tr^X[X_2] + tr^X[X_1]$$

Transport parameter

The values of the transport parameters that make the steady state of the compartmental model exactly match the PDE averages may then be computed as follows:

$$tr^X = \frac{k_0 - k_1\langle X\rangle_1}{\langle X\rangle_1 - \langle X\rangle_2},$$

Case 4: Compartmentalized two-site modification (Mass-action)

We consider the system involving interconversion between the species X and its modified forms, X^* and X^{**}, with the three forms belonging to a conserved pool. The phosphorylation reactions are assumed to be in the mass action regime, with kinases restricted to compartment 1 and phosphatases in compartment 2. All species are assumed to be equally diffusible.

1-D PDE Model

In compartment 1 ($0 \leq \theta \leq L_1$),

$$\frac{\partial[X]}{\partial t} = -k_1[X] + D_X\frac{\partial^2[X]}{\partial\theta^2}$$
$$\frac{\partial[X^*]}{\partial t} = k_1[X] - k_2[X^*] + D_X\frac{\partial^2[X^*]}{\partial\theta^2}$$
$$\frac{\partial[X^{**}]}{\partial t} = k_2[X^*] + D_X\frac{\partial^2[X^{**}]}{\partial\theta^2}$$

Between the compartments ($L_1 \leq \theta \leq L_1 + L$),

$$\frac{\partial [X]}{\partial t} = D_X \frac{\partial^2 [X]}{\partial \theta^2}$$
$$\frac{\partial [X^*]}{\partial t} = D_X \frac{\partial^2 [X^*]}{\partial \theta^2}$$
$$\frac{\partial [X^{**}]}{\partial t} = D_X \frac{\partial^2 [X^{**}]}{\partial \theta^2}$$

In compartment 2 ($L_1 + L \leq \theta \leq L_1 + L + L_2 = l$),

$$\frac{\partial [X]}{\partial t} = k_4 [X^*] + D_X \frac{\partial^2 [X]}{\partial \theta^2}$$
$$\frac{\partial [X^*]}{\partial t} = k_3 [X^{**}] - k_4 [X^*] + D_X \frac{\partial^2 [X^*]}{\partial \theta^2}$$
$$\frac{\partial [X^{**}]}{\partial t} = -k_3 [X^{**}] + D_X \frac{\partial^2 [X^{**}]}{\partial \theta^2}$$

With the concentration profiles satisfying a conservation condition of the form,

$$\frac{X^{Total}}{l} = [X] + [X^*] + [X^{**}] \; for \; 0 \leq \theta \leq l$$

1-D PDE Solution

$$
\begin{aligned}
For \; 0 \leq \theta < L_1: \; [X] &= X_T c_1 \cosh(\omega_1 \theta) \\
[X^*] &= X_T \left(c_2 \cosh(\omega_2 \theta) - \frac{k_1 c_1}{k_1 - k_2} \cosh(\omega_1 \theta) \right) \\
[X^* *] &= X_T - [X] - [X^*] \\
For \; L_1 \leq \theta < L_1 + L: \; [X] &= X_T (c_3 \theta + c_4) \\
[X^*] &= X_T (c_5 \theta + c_6) \\
[X^* *] &= X_T - [X] - [X^*] \\
For \; L_1 + L \leq \theta < l: \; [X] &= X_T - [X^*] - [X^{**}] \\
[X^*] &= X_T \left(c_8 \cosh(\omega_4 (l - \theta)) - \frac{k_3 c_7}{k_3 - k_4} \cosh(\omega_3 (l - \theta)) \right) \\
[X^{**}] &= X_T c_7 \cosh(\omega_3 (l - \theta)) \\
where \; l &= L_1 + L + L_2; \\
\omega_1^2 &= \frac{k_1}{D_X}; \; \omega_2^2 = \frac{k_2}{D_X}; \; \omega_3^2 = \frac{k_3}{D_X}; \; \omega_4^2 = \frac{k_4}{D_X}
\end{aligned}
$$

The constants c_i ($i = 1, ..., 8$) that determine the solution, are obtained by using the boundary conditions - no-flux conditions at the outer boundaries of the domain, and continuity of the concentration profiles and their first derivatives, at the compartment boundaries. This yields eight linear equations in the variables c_i, which are then solved by symbolic computation using Maple. The expressions thus obtained for the steady state concentration profiles of $[X]$, $[X^*]$, and $[X^{**}]$ are then validated by comparison with the steady state profiles obtained by numerical solution of the PDE system.

2.5 Thin compartment regime in a 2-D system

We have seen that a compartmental model, with fixed transport parameters of the form D/LL_i, is a good approximation for a 1-D system in the thin compartment regime, at steady state. Here we show how a similar ODE model may be obtained for the same kind of system in a general 2-D case, with smooth boundaries for the compartments and the spatial domain. Consider the 2-D system shown in Fig. S1(b). Since the kinetics play no role in representing transport in the thin compartment regime, we can focus on a single species X to get the general picture. At steady state, let $X(\vec{r})$ denote the concentration of species X outside the compartments. Within the compartments, and on the compartment boundaries (Γ_1 and Γ_2), the concentrations are given by the compartmental averages - X_1 and X_2.

Since the intermediate space involves only diffusion of X, at steady state we have

$$\begin{aligned}
&\nabla^2 X(\vec{r}) = 0 \\
&\quad \textit{with the boundary conditions} \\
&\nabla X(\vec{r}) = 0 \;\; \textit{on the outer boundary}\ \Gamma \;\; (\textit{no-flux}) \\
&X(\vec{r}) = X_1 \;\; \textit{on the boundary}\ \Gamma_1 \\
&X(\vec{r}) = X_2 \;\; \textit{on the boundary}\ \Gamma_2
\end{aligned}$$

The solution to this boundary value problem may be expressed as

$$X(\vec{r}) = X_1 f_1(\vec{r}) + X_2 f_2(\vec{r})$$

where the functions $f_1(\vec{r})$ and $f_2(\vec{r})$ are solutions to the above boundary problem for the basal cases $X_1 = 1,\ X_2 = 0$ and $X_1 = 0,\ X_2 = 1$ respectively. As long as the geometry of the system remains unchanged, these functions only need to be solved for once (perhaps numerically), and can be used in the following way to obtain a compartmental description in the thin compartment regime. Note that the gradient of $X(\vec{r})$ is given by

$$\nabla X(\vec{r}) = X_1 \nabla f_1(\vec{r}) + X_2 \nabla f_2(\vec{r}) \tag{13}$$

At steady state, the net transport of X out of compartment 1 is given by averaging the flux over the boundary Γ_1:

$$Net\ Transport = -D_X \int_{\Gamma_1} \nabla X(\vec{r}).\hat{n}ds,$$

where $\hat{n}$ is the unit outward normal to Γ_1. This can be rewritten as:

$$Net\ Transport = -D_X \left(\int_{\Gamma_1} \nabla f_1(\vec{r}).\hat{n}ds \right) X_1 - D_X \left(\int_{\Gamma_1} \nabla f_2(\vec{r}).\hat{n}ds \right) X_2, \tag{14}$$

In the compartmental model for this 2-D system, the net transport of X out of compartment 1 is represented by:

$$Net\ Transport = A_1 tr^X_{1\to 2} X_1 - A_2 tr^X_{2\to 1} X_2 \tag{15}$$

where X_i are the compartmental (average) concentrations and A_i are the volumes (areas) of the compartments. Comparing (14) and (15), we see that the two transport parameters associated with the species X in the compartmental model are given by:

$$tr^X_{1\to 2} = -\frac{D_X}{A_1} \left(\int_{\Gamma_1} \nabla f_1(\vec{r}).\hat{n}ds \right),$$
$$tr^X_{2\to 1} = \frac{D_X}{A_2} \left(\int_{\Gamma_1} \nabla f_2(\vec{r}).\hat{n}ds \right)$$

2.6 Finding a compartmental ODE description to match the PDE

1-D PDE at steady state

Consider a general pathway with first-order mass-action kinetics and possible zeroth order production, distributed between two equally sized compartments ($L_1 = L_2$), in a 1-D domain (see Figure 1 for the geometry). At steady state, the PDE model in vector form gives:

$$A_1 u + b_1 + D\frac{\partial^2 u}{\partial \theta^2} = 0\ in\ compartment\ 1$$
$$D\frac{\partial^2 u}{\partial \theta^2} = 0\ in\ the\ intermediate\ space$$
$$A_2 u + b_2 + D\frac{\partial^2 u}{\partial \theta^2} = 0\ in\ compartment\ 2 \tag{16}$$

Here, u is the vector of species concentrations at a point in the domain, and A_1 and A_2 are square matrices containing the rate constants corresponding to the mass-action reactions in compartments 1 and 2 respectively. The vectors b_1 and b_2 represent possible zeroth order production terms, and D is a diagonal matrix containing the diffusivities of the different species. For example, for the single modification cycle discussed in the main text, with kinetics at the mass-action regime,

$$
\begin{aligned}
A_1 = \begin{bmatrix} -k_1 & 0 \\ k_1 & 0 \end{bmatrix};\ A_2 &= \begin{bmatrix} 0 & k_2 \\ 0 & -k_2 \end{bmatrix} \\
b_1 = b_2 &= \begin{bmatrix} 0 \\ 0 \end{bmatrix} \\
D &= \begin{bmatrix} D_X & 0 \\ 0 & D_{X^*} \end{bmatrix}
\end{aligned}
$$

No-flux boundaries are present at the ends of the domain. Averaging over compartments 1 and 2 at steady state, we have

$$
A_1\langle u\rangle_1 + b_1 + \frac{1}{L_1} D \left(\frac{\partial u}{\partial \theta}\right)_{L_1} = 0 \tag{17}
$$

$$
A_2\langle u\rangle_2 + b_2 - \frac{1}{L_1} D \left(\frac{\partial u}{\partial \theta}\right)_{L_1+L} = 0 \tag{18}
$$

Here, $\langle u\rangle_1$ and $\langle u\rangle_2$ are vectors of compartmental averages for compartments 1 and 2. Since no reactions occur in the intermediate space, we also have

$$
\left(\frac{\partial u}{\partial \theta}\right)_{L_1} = \left(\frac{\partial u}{\partial \theta}\right)_{L_1+L} \tag{19}
$$

This also means that

$$
A_1\langle u\rangle_1 + b_1 = -A_2\langle u\rangle_2 - b_2 \tag{20}
$$

Compartmental ODE

For the system described by (16), we consider a compartmental ODE model where each species has a single transport parameter associated with it. The compartmental model has the form:

$$
\begin{aligned}
\frac{d\bar{u}_1}{dt} &= A_1\bar{u}_1 + b_1 + \Delta\sigma \\
\frac{d\bar{u}_2}{dt} &= A_2\bar{u}_2 + b_2 - \Delta\sigma
\end{aligned} \tag{21}
$$

Here, $\bar{u}_1$ and $\bar{u}_2$ denote vectors of species concentrations corresponding to compartments 1 and 2 respectively. σ is a vector containing the transport parameters, and Δ is a diagonal matrix having the components of the vector $\bar{u}_2 - \bar{u}_1$ as its diagonal.

Since we want the compartmental model to match the PDE compartmental averages at steady state, the steady state equations for the compartmental model must satisfy the equations:

$$A_1\langle u\rangle_1 + b_1 + \Delta_u\sigma = 0$$
$$A_2\langle u\rangle_2 + b_2 - \Delta_u\sigma = 0$$

where Δ_u is a diagonal matrix having the vector $\langle u\rangle_2 - \langle u\rangle_1$ as its diagonal. Thus, the matrix Δ_u is non-singular, assuming that the system corresponds to a case where all diffusing species exhibit gradients between compartments. This fact, along with (20), allows us to compute the unique vector of transport parameters- σ_{ss}, that produces an exact match between the compartmental model and the PDE averages at steady state.

$$\sigma_{ss} = -\Delta_u^{-1}(A_1\langle u\rangle_1 + b_1) \tag{22}$$

Note that there are exceptional cases where the compartmental averages may be equal for certain species (i.e. certain components of the vector $\langle u\rangle_2 - \langle u\rangle_1$ may be zero), even when there is a non-zero concentration gradient for this species between compartments. In such cases, the transport parameter associated with this species diverges (See main text).

Of course, in addition to using these transport parameters, the ability of the compartmental model to match the PDE at steady state also relies on correctly describing the conservation conditions for any conserved species, as seen previously.

Compartmental ODEs: Nonlinear kinetics

We now examine the case similar to (16), but where the kinetics can have arbitrary form, i.e. possibly including nonlinear terms such as second-order mass action steps or Michaelis-Menten kinetics. At steady state we have:

$$f_1(u) + D\frac{\partial^2 u}{\partial \theta^2} = 0 \textit{ in compartment } 1$$
$$D\frac{\partial^2 u}{\partial \theta^2} = 0 \textit{ in the intermediate space}$$
$$f_2(u) + D\frac{\partial^2 u}{\partial \theta^2} = 0 \textit{ in compartment } 2 \tag{23}$$

where $f_1(u)$ and $f_2(u)$ are nonlinear, vector valued functions of u representing the sum of the kinetic terms in compartments 1 and 2. Averaging over the compartments, we find

$$\langle f_1(u)\rangle_1 + \frac{1}{L_1} D \left(\frac{\partial u}{\partial \theta}\right)_{L_1} = 0$$
$$\langle f_2(u)\rangle_2 - \frac{1}{L_1} D \left(\frac{\partial u}{\partial \theta}\right)_{L_1+L} = 0$$

As before, we have

$$\langle f_1(u)\rangle_1 = -\langle f_2(u)\rangle_2$$

Note however, that these are spatial averages of $f_1(u)$ and $f_2(u)$, and not functions of the average of u, i.e. $f_1(\langle u\rangle_1)$ and $f_2(\langle u\rangle_2)$. In general, these are not equal, and therefore,

$$f_1(\langle u\rangle_1) \neq -f_2(\langle u\rangle_2) \tag{24}$$

Now consider the compartmental model for the system (23), given by

$$\frac{d\bar{u}_1}{dt} = f_1(\bar{u}_1) + \Delta\sigma$$
$$\frac{d\bar{u}_2}{dt} = f_2(\bar{u}_2) - \Delta\sigma \tag{25}$$

At steady state, the vectors of compartmental concentrations, $\bar{u}_1$ and $\bar{u}_2$ must satisfy

$$f_1(\bar{u}_1) = -f_2(\bar{u}_2) \tag{26}$$

From (24) and (26), we can conclude that the compartmental model cannot exactly match the PDE averages at steady state, when nonlinear kinetic terms are involved. The best that can be done in this case is to choose the vector of transport parameters such that the mismatch is minimized in some sense. In the present study, we choose to minimize the sum-squared error $\|(\bar{u}_1 - \langle u\rangle_1)\|^2 + \|(\bar{u}_2 - \langle u\rangle_2)\|^2$. For the compartmental model (10), this gives us a nonlinear optimization problem with a quadratic objective function and nonlinear equality constraints:

$$\begin{aligned} \min_{\sigma} \quad & \|(\bar{u}_1 - \langle u\rangle_1)\|^2 + \|(\bar{u}_2 - \langle u\rangle_2)\|^2 \\ \text{s.t.} \quad & f_1(\bar{u}_1) + \Delta\sigma = 0 \\ & f_2(\bar{u}_2) - \Delta\sigma = 0 \end{aligned} \tag{27}$$

Any conservation conditions would form additional equality constraints in (27).

Transport Parameters and Diffusivities

We now examine the possibility that, for the first-order mass-action system in (16), the transport parameters for the different species, that produce an exact steady state match, are in the same ratio as the diffusivities of the corresponding species. For this to be true, at steady state we need the vectors of compartmental averages, $\langle u \rangle_1$ and $\langle u \rangle_2$ to satisfy an equation of the form:

$$A_1 \langle u \rangle_1 + b_1 + \lambda D(\langle u \rangle_2 - \langle u \rangle_1) = 0 \tag{28}$$

for some positive λ. Thus, from equations (17) and (28), we need

$$\frac{1}{L_1}\left(\frac{\partial \bar{u}}{\partial \theta}\right)_{L_1} = \lambda(\langle \bar{u} \rangle_2 - \langle \bar{u} \rangle_1) \tag{29}$$

Single modification cycle (X to X*). Since the kinetics in each compartment conserves the total amount of species, the species concentrations satisfy

$$D_X X + D_{X^*} X^* = X_T \tag{30}$$

at all points in the domain. X_T is constant within the domain but its value depends on both kinetic and spatial parameters (including compartment size, compartment separation, and diffusivities). Thus, the compartmental averages satisfy,

$$D_X \langle X \rangle_1 + D_{X^*} \langle X^* \rangle_1 = D_X \langle X \rangle_2 + D_{X^*} \langle X^* \rangle_2 = X_T$$

Rewriting this,

$$D_X \left(\langle X \rangle_1 - \langle X \rangle_2\right) + D_{X^*} \left(\langle X^* \rangle_1 - \langle X^* \rangle_2\right) = 0 \tag{31}$$

Now, the steady state mass balance for compartment 1 implies

$$D_X \left(\frac{\partial X}{\partial \theta}\right)_{L_1} + D_X^* \left(\frac{\partial X^*}{\partial \theta}\right)_{L_1} = 0 \tag{32}$$

i.e. the net flux out of the compartment is zero.

Equations (31) and (32) imply that the differences in compartmental averages are related to the concentration gradients in the intervening space, as follows

$$\begin{bmatrix} \left(\frac{\partial X}{\partial \theta}\right)_{L_1} \\ \left(\frac{\partial X^*}{\partial \theta}\right)_{L_1} \end{bmatrix} = \gamma \begin{bmatrix} \langle X \rangle_1 - \langle X \rangle_2 \\ \langle X^* \rangle_1 - \langle X^* \rangle_2 \end{bmatrix} \tag{33}$$

Thus, the compartmental fluxes and compartmental averages satisfy an equation of the form (29). This means that a compartmental ODE model that exactly matches the PDE compartmental averages at steady state, must have transport parameters in the same ratio as the diffusivites. For a given set of kinetic and spatial parameters, these transport parameters would be uniquely determined.

Two-site modification cycle (X to X* to X).** Since the kinetics in each compartment only results in an interconversion between species, adding the equations for X, X^*, and X^{**} at steady state results in:

$$D_X X + D_{X^*} X^* + D_{X^{**}} X^{**} = X_T \tag{34}$$

at all points in the domain, where X_T is constant across the domain As in the previous case, the compartmental averages for the different species satisfy

$$D_X \left(\langle X\rangle_1 - \langle X\rangle_2\right) + D_{X^*} \left(\langle X^*\rangle_1 - \langle X^*\rangle_2\right) + D_{X^{**}} \left(\langle X^{**}\rangle_1 - \langle X^{**}\rangle_2\right) = 0 \tag{35}$$

Considering the steady state mass balance for compartment 1, we have

$$D_X \left(\frac{\partial X}{\partial \theta}\right)_{L_1} + D_X^* \left(\frac{\partial X^*}{\partial \theta}\right)_{L_1} + D_X^{**} \left(\frac{\partial X^{**}}{\partial \theta}\right)_{L_1} = 0 \tag{36}$$

However, unlike the case of the single modification cycle, equations (35) and (36) do not suffice to give us a relationship of the form

$$\begin{bmatrix} \left(\frac{\partial X}{\partial \theta}\right)_{L_1} \\ \left(\frac{\partial X^*}{\partial \theta}\right)_{L_1} \\ \left(\frac{\partial X^{**}}{\partial \theta}\right)_{L_1} \end{bmatrix} = \gamma \begin{bmatrix} \langle X\rangle_1 - \langle X\rangle_2 \\ \langle X^*\rangle_1 - \langle X^*\rangle_2 \\ \langle X^{**}\rangle_1 - \langle X^{**}\rangle_2 \end{bmatrix} \tag{37}$$

This means that a compartmental ODE, which matches the PDE compartmental averages at steady state, need not have transport parameters in the same ratio as the diffusivities. However, the transport parameter associated with each species would still be uniquely determined. The transport parameters for the exact match, obtained numerically (solving the PDE and computing compartmental averages) for a sample case, are shown in Fig. 5(b).

3 Additional Information

3.1 Adding a third compartment

We examine the case of adding a third compartment to an existing two compartment open pathway (see schematic in Fig. 4(c)). The three compartments are of the same size, and are equally spaced. The species X is produced at a constant rate (zeroth order kinetics) in compartment 1, and diffuses across the domain. X is consumed by reactions in compartments 1, 2 and 3 (represented by first order reactions). We obtain the analytical solution to this three compartment system at steady state (not shown), and use the steady state concentration profile of X to compute spatial averages within each compartment. We use these averages to calculate the two transport parameters tr_{12}^X and tr_{23}^X representing transport between compartments 1 and 2 and between 2 and 3 respectively, as shown in (22) for the general case. Each of these transport parameters depend on the diffusivity, compartment sizes, compartment separation, and all the kinetic parameters. We fix the diffusivity, compartment sizes, compartment separation, and the kinetic parameters in compartments 1 and 2 (see table below for parameter values), and examine the effect of varying the rate constant of X consumption in compartment 3. The result is shown in Fig. 4(d). Note that the analytical solution to the three compartment system was cross-validated by comparison to the numerical solution of the PDE.

3.2 Bistable two-site modification

As discussed in the text, we consider a compartmentalized two-site modification pathway (see Fig. 5(a)), in a kinetic regime where it exhibits two stable steady states, over a range of values of E_1^{Total} - the total amount of kinase in compartment 1. We consider this system in the thin compartment regime ($L/L_1 = 18$). The concentration profiles for the two steady states are obtained by numerical solution of the PDE model, with a fixed total amount of substrate $X^{Total} = 10$ and enzymes $E_1^{Total} = 0.6$; $E_2^{Total} = 5$. We note that the total amount of substrate (free substrate + complexes) in the compartments is different for the two steady states. Now, for a given steady state, there are two ways of calibrating the compartmental model with the correct total amount of substrate in the compartment. One way is to obtain this total amount from the concentration profiles of the given steady state. However, since this amount is different for the two steady states, the resulting compartmental description can only capture one of these accurately (i.e. the steady state used for the calibration). As shown in Fig. 5(f) this may not

even capture the second steady state. The other way is to use the modified conservation condition discussed previously, which accounts for substrate in intermediate space as well. This is a good approximation because the system is in the thin compartment regime. By using this conservation condition, the compartmental model is able to accurately capture both steady states.

Figure S1

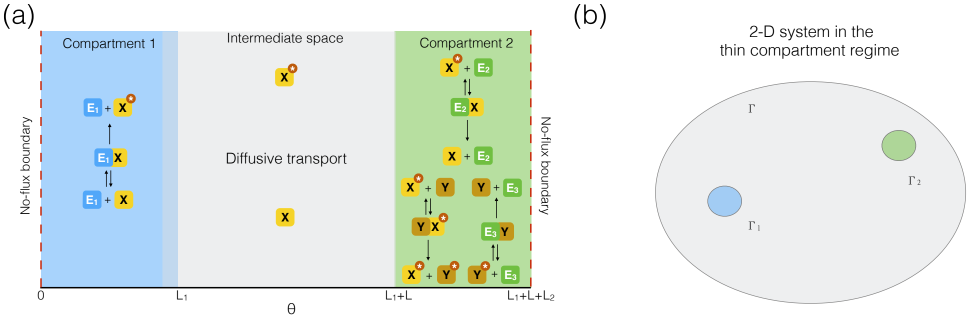

(a)
Compartment 1
Intermediate space
Compartment 2
No-flux boundary
Diffusive transport
No-flux boundary
0
L₁
L₁+L
L₁+L+L₂
θ
(b)
2-D system in the
thin compartment regime
Γ
Γ₂
Γ₁

Parameter values

Figure 1 (b)	Total_amount of substrate = 2<br>$k_1 = 1$<br>$k_2 = 2$<br>$D_X = 0.05$<br>$D_{X^*} = 0.1$<br>L/L1 = 18<br>Domain size = 1
Figure 1(c)	Total_amount of substrate = 2<br>$k_1 = 1$<br>$k_2 = 2$<br>$D_X = 0.05$<br>$D_{X^*} = 0.1$<br>L/L1 = 18<br>Domain size = 1
Figure 1(d)	Total_amount of substrate = 10<br>$k_1 = 10$<br>$k_{-1} = 0.1$<br>$k_2 = 1.5$<br>$k_3 = 2$<br>$k_{-3} = 0.2$<br>$k_4 = 0.4$<br>$E_1^{Total} = 10$<br>$E_2^{Total} = 1\ (increased\ to\ 10)$<br>$D_X = D_{X^*} = 0.1$<br>L/L1 = 18<br>Domain size = 1<br>**Note:** The sequestration in the intermediate space is accounted for exactly, in both the basal and perturbed cases, using the numerical solution of the PDE.
Figure 2(a), (b)	Total_amount of substrate = 2<br>$k_1 = 1$ (basal); $k_1 = 100$ (perturbed)<br>$k_2 = 2$<br>$D_X = 0.05$<br>$D_{X^*} = 0.1$<br>L/L1 = 0.5<br>Domain size = 1<br>**Note:** The sequestration in the intermediate space is accounted for exactly, in both the basal and perturbed cases, using the analytical solution.
Figure 2(c)	Total_amount of substrate = 10<br>$k_1 = 1$ (basal); $k_1 = 100$ (perturbed)<br>$k_{-1} = 0.1$<br>$k_2 = 300$

	$k_3 = 2$<br>$k_{-3} = 0.2$<br>$k_4 = 400$<br>$E_1^{Total} = 1$<br>$E_2^{Total} = 1$<br>$D_X = D_{X^*} = 0.1$<br><br>L/L1 = 0.5<br>Domain size = 1<br><br>D/(L1*L) = 1.25<br>Transport parameter = 0.54 (basal); 0.68 (perturbed)<br>(Computed by minimizing error)<br>**Note:** The sequestration in the intermediate space is accounted for exactly, in both the basal and perturbed cases, using the numerical solution of the PDE.
Figure 3(a)	Total amount of substrate = 2<br>$k_2 = 2$<br>$D_X = 0.1$<br>$D_{X^*} = 0.1$<br>L/L1 = 1, 0.1<br>Domain size = 1
Figure 3(b)	Total amount of substrate = 2<br>$k_1 = 10$<br>$k_2 = 2$<br>$D_X = 0.1$<br>$D_{X^*} = 0.1$<br>L/L1 = 18<br>Domain size = 1
Figure 3(c) and Figure 3(d)	Total amount of substrate = 2<br>$k_1 = 10$<br>$k_2 = 2$<br>$D_X = 0.1$<br>$D_{X^*} = 0.1$<br>Vary L, keeping D/L constant<br>Domain size = 1
Figure 4(b) (Simple open pathway)	$k_0 = 1$<br>$D_X = 0.1$<br>L/L1 = 1, 0.1<br>Domain size = 1
Figure 4(d) (Three compartment open system)	$k_0 = 1$ (Compartment 1)<br>$k_1 = 1$ (Compartment 1)<br>$k_2 = 0.1$ (Compartment 2)<br>$D_X = 0.1$<br>Equal sized compartments, L1=L2=L3=1/3

	Equally spaced, separation = 1/15
Figure 5(b) (Two-site modification)	Mass action limit<br>$k_1 = 1.5$<br>$k_2 = 0.02$<br>$k_3 = 0.1$<br>$k_4 = 2$<br>$D_X = D_{X^*} = D_{X^{**}} = 0.01$<br>Domain size = 1<br>L/L1 = 0.5
Figure 5(c) (Two-site modification)	Mass action limit<br>$k_1 = 1.5$<br>$k_2 = 2$<br>$k_3 = 0.05$<br>$k_4 = 1$<br>$D_X = D_{X^*} = D_{X^{**}} = 0.01$<br>Domain size = 1<br>L/L1 = 0.5
Figure 5(d) (Two-site modification)	Mass action limit<br>$k_1 = 1.5$<br>$k_2 = 2$<br>$k_3 = 0.05$<br>$D_X = D_{X^*} = D_{X^{**}} = 0.01$<br>Domain size = 1<br>L/L1 = 0.5
Figure 5 (e) and (f)	Total amount in whole domain = 10<br>$k_1 = 45$<br>$k_{-1} = 0.5$<br>$k_2 = 4$<br>$k_3 = 125$<br>$k_{-3} = 0.5$<br>$k_4 = 80$<br>$k_5 = 15$<br>$k_{-5} = 0.05$<br>$k_6 = 1$<br>$k_7 = 25$<br>$k_{-7} = 0.05$<br>$k_8 = 3.5$<br><br>$E_1^{Total} = 0.6\ (basal\ value)$<br>$E_2^{Total} = 5$<br><br>$D_X = D_{X^*} = D_{X^{**}} = 0.1$<br>L/L1 = 18<br>Domain size = 1
Figure 6(b)	$k_1 = 0.02$<br>$k_{21} = 0.1$<br>$k_{22} = 1$<br>$k_3 = 0.5$

	$E^{Total} = 1$<br>$F^{Total} = 1$<br>$D_X = 0.05$<br><br>L1=L2=2<br>L=0.1
Figure 6(c)	$k_1 = 0.02$<br>$K = 0.01$<br>$k_{21} = 0.1$<br>$k_{22} = 10$<br>$k_3 = 0.001$<br>$E^{Total} = 1$<br>$F^{Total} = 2$<br>$D_X = 0.1$<br>L1=L2=9/20<br>L=0.1;
